# Evidence That Tumor Microenvironment Initiates Epithelial-To-Mesenchymal Transition and Calebin A can Suppress it in Colorectal Cancer Cells

**DOI:** 10.3389/fphar.2021.699842

**Published:** 2021-07-02

**Authors:** Constanze Buhrmann, Aranka Brockmueller, Choudhary Harsha, Ajaikumar B. Kunnumakkara, Peter Kubatka, Bharat B. Aggarwal, Mehdi Shakibaei

**Affiliations:** ^1^ Musculoskeletal Research Group and Tumor Biology, Chair of Vegetative Anatomy, Faculty of Medicine, Institute of Anatomy, Ludwig-Maximilian-University Munich, Munich, Germany; ^2^ Faculty of Medicine, Institute of Anatomy and Cell Biology, University of Augsburg, Augsburg, Germany; ^3^ Cancer Biology Laboratory and DBT-AIST International Center for Translational and Environmental Research (DAICENTER), Department of Biosciences and Bioengineering, Indian Institute of Technology Guwahati, Guwahati, India; ^4^ Department of Medical Biology, Jessenius Faculty of Medicine, Comenius University in Bratislava, Martin, Slovakia; ^5^ Inflammation Research Center, San Diego, CA, United States

**Keywords:** tumor microenvironment, colorecal cancer, Calebin A, EMT, focal adhesion kinase, NF- kappa B, slug, TGF - β1

## Abstract

**Background:** Tumor microenvironment (TME) has a pivotal impact on tumor progression, and epithelial-mesenchymal transition (EMT) is an extremely crucial initial event in the metastatic process in colorectal cancer (CRC) that is not yet fully understood. Calebin A (an ingredient in *Curcuma longa*) has been shown to repress CRC tumor growth. However, whether Calebin A is able to abrogate TME-induced EMT in CRC was investigated based on the underlying pathways.

**Methods:** CRC cell lines (HCT116, RKO) were exposed with Calebin A and/or a FAK inhibitor, cytochalasin D (CD) to investigate the action of Calebin A in TME-induced EMT-related tumor progression.

**Results:** TME induced viability, proliferation, and increased invasiveness in 3D-alginate CRC cultures. In addition, TME stimulated stabilization of the master EMT-related transcription factor (Slug), which was accompanied by changes in the expression patterns of EMT-associated biomarkers. Moreover, TME resulted in stimulation of NF-κB, TGF-β1, and FAK signaling pathways. However, these effects were dramatically reduced by Calebin A, comparable to FAK inhibitor or CD. Finally, TME induced a functional association between NF-κB and Slug, suggesting that a synergistic interaction between the two transcription factors is required for initiation of EMT and tumor cell invasion, whereas Calebin A strongly inhibited this binding and subsequent CRC cell migration.

**Conclusion:** We propose for the first time that Calebin A modulates TME-induced EMT in CRC cells, at least partially through the NF-κB/Slug axis, TGF-β1, and FAK signaling. Thus, Calebin A appears to be a potential agent for the prevention and management of CRC.

## Introduction

Colorectal cancer (CRC) is ranked third in incidence and second in associated mortality worldwide (Bray et al., 2018; Bray et al., 2020; Siegel et al., 2020). CRC rising incidences have been linked to the aging population, poor lifestyle, and diet in particular, among other factors (Dekker et al., 2019). Although, it has been observed that the incidence of CRC has been decreasing in recent years (Mauri et al., 2019), additionally it has been reported that the quality of life is often compromised by chronic side effects in CRC patients after therapies (Buccafusca et al., 2019). This indicates the importance of developing an alternative effective treatment option with few side effects in order to control the overall CRC mortality statistics.

The major etiological factor of CRC is chronic infection in the gastrointestinal tract that causes an active pro-inflammatory tumor microenvironment (TME) supported by defensive cells such as T-lymphocytes, macrophages, natural killer cells and other cells that may play a key role in tumor development (Elinav et al., 2013). The TME contributes significantly to the proliferation, survival, and migration of the underlying cellular neoplastic system. In addition, tumor cells possess some of the signaling proteins of the inherent immune system, such as chemokines, cytokines and their receptors that contribute to migration and metastasis (Coussens and Werb, 2002). Further, the inflammatory TME attracts immune cells and other protective cells which secrete pro-inflammatory mediators. These factors, in turn, can lead to greater alteration of intracellular signaling pathways, initiation and accumulation of mutations in tumor cells that induce active and rapid proliferation and protect the cancer cells from apoptosis. As a result, CRC cells lose the epithelial phenotype and simultaneously acquire the mesenchymal phenotype, thus undergoing an epithelial-mesenchymal transition (EMT) that gives them the ability to metastasize (Virchow, 1881; Coussens and Werb, 2002; de Visser et al., 2006; Medzhitov, 2008; Buhrmann et al., 2020a). Therefore, targeting the TME might provide new therapeutic approaches for controlling tumor development.

EMT and its associated progression and occurrence of metastasis is a major contributor to mortality in CRC patients, and the underlying mechanisms are still under exploration. EMT has been shown to play a determinant function in cancer development as it leads to disruption of cell-cell adherences, extracellular matrix (ECM) re-modelling and increased motility of the tumor cells (Martin, 2014). In addition, EMT inhibits E-cadherin expression, causing impaired adhesion and cell polarity during the development of epithelial tumors (carcinomas) and becomes evident at the stage of invasion (mesenchymal phenomenon), when tumor cells detach from the primary tumor and acquire the ability to cross the surrounding area (Behrens et al., 1992; Stetler-Stevenson et al., 1993; Christofori and Semb, 1999; Tepass et al., 2000; Thiery, 2002; Prieto-García et al., 2017) and this promotes formation of cancer stem cells (Mani et al., 2008; Polyak and Weinberg, 2009). Interestingly, it has been previously reported that down-regulation of E-cadherin expression in tumor cells may involve both genetic and epigenetic modifications (Christofori and Semb, 1999). Among the epigenetic and genetic modifications, excessive methylation of the E-cadherin promoter and transcriptional suppression are observed as major activities in most carcinomas (Yoshiura et al., 1995; Tamura et al., 2000; Cheng et al., 2001; Hajra et al., 2002). In addition, transcriptional repressors of the Snail zinc finger transcription factor family, such as Snail and Slug, play an essential role in the activation and induction of EMT, as this alters the expression patterns of EMT biomarkers in favor of the mesenchymal phenotype (LaBonne and Bronner-Fraser, 2000; del Barrio and Nieto, 2002; Nieto, 2002). Indeed, the inflammatory TME has been further shown to induce cancer initiation, and metastasis through stimulating the EMT process (Buhrmann et al., 2016). Stromal cells in the TME are recognized to have a pivotal function in the stimulation of EMT in tumor cells (Guo and Deng, 2018) and TME-mediated EMT induction in tumor cells is stimulated by activating various cell signaling pathways including TNF-β, NF-κB and Wnt signaling connected with cancer progression (Jing et al., 2011). Moreover, pro-inflammatory TNF-α or TNF-β, have been previously reported to act as potential endogenous tumor-promoting factors and an important mechanism of cytokines in this action is to induce EMT in different types of tumor cells (Soria et al., 2011; Buhrmann et al., 2020a). In addition, focal adhesion kinase (FAK), cytoskeletal proteins and their receptors have previously demonstrated to have a major function in the context of EMT-mediated tumor cell survival and invasion, as well as progression. These upstream signaling molecules are potential therapeutic targets to control tumor metastasis as well as tumor proliferation (Seguin et al., 2015).

NF-κB is a very important and pro-inflammatory transcription factor that is induced by diverse inflammatory signals, oxidative stress, and various carcinogens in diverse cells. Moreover, NF-κB is known to initially reside in an inactive state in the cytoplasm in various cells and consists of a heterotrimer with p50 and p65 subunits and an inhibitory IκBα subunit (Bhat-Nakshatri et al., 2010). It has been shown that most of the processes involved in pro-inflammatory and carcinogenic pathways are linked to NF-κB signaling. After activation and phosphorylation, NF-κB translocates to the nucleus to control the protein expression of pro-inflammatory genes. Interestingly, it is found that phosphorylation of NF-κB leads to various stages of inflammation, survival, proliferation, migration, angiogenesis, tumor development, and also tumor cell metastasis and drug resistance (Aggarwal, 2004).

The transforming growth factor-β1 (TGF-β1) is identified to be a potent enabler of EMT in the initial phase of tumor progression and tumor metastasis (Buhrmann et al., 2016). In addition, TGF-β1 specifically promotes receptor complex activity, leading to the initiation of Smad2 phosphorylation in the nucleus, in which they functionally associate to transcription factors such as Snail and Slug, suppressing the expression of epithelial biomarkers and stimulating the expression of mesenchymal biomarkers at mRNA level (Zhang et al., 2018).

Further, activation of EMT signaling pathways has been described to be regulated at the epigenetic and post-translational level (Prieto-García et al., 2017) and identification of novel agents targeting EMT may provide possible new therapeutic approaches in cancer treatment (Kalluri and Weinberg, 2009). Calebin A [4-(3-methoxy-4-hydroxyphenyl)-2-oxo-3-enebutanyl 3-(3-methoxy-4-hydroxyphenyl) propenoate] is drived from curcumin-free turmeric (*Curcuma longa L., Zingiberaceae*), first described by Park and Kim (Park and Kim, 2002) and since then, other isomers of Calebin A have been discovered (Zeng et al., 2007). Calebin A has been reported to possess potent anti-inflammatory and anti-tumor potential, such as inhibiting cell viability, proliferation, and migration, and promoting apoptosis in various resistant and solid tumor cells by blocking several cellular signaling pathways (Aggarwal et al., 2013; Buhrmann et al., 2019; Nair et al., 2019; Buhrmann et al., 2020a).

However, so far, to our knowledge, there are not any clear studies published on the possible inhibition of EMT process and metastasis in CRC-TME by Calebin A. Therefore, the present study targeted to test the sub-regulatory promotions of Calebin A on CRC cells in a pro-inflammatory multicellular TME model. In this work, we report for the first time that Calebin A suppresses TME-induced invasion and malignancy of CRC cells by modulating EMT signaling pathways, thus highlighting Calebin A.

## Materials and Methods

### Antibodies and Chemicals

Monoclonal antibodies to phospho-specific-FAK, PARP, p65-NF-κB, phospho-specific p65-NF-κB, and activated-Caspase-3 were from R&D Systems (Heidelberg, Germany). Antibodies to β-Actin, cytochalasin D (CD), MTT reagent [3-(4,5-dimethylthiazol-2-yl)-2,5-diphenyltetrazolium bromide], DAPI (4,6-diamidino-2-phenylindole), dithiothreitol (DTT) and alginate were from Sigma-Aldrich (Taufkirchen, Germany). Anti-E-cadherin, anti-vimentin, anti-TGF-β1, anti-p-Smad2 and anti-Slug were from Santa Cruz Biotechnology (Santa Cruz, CA, United States). Secondary antibodies for immunofluorescence were procured from Dianova (Hamburg, Germany). Alkaline phosphatase-linked antibodies for Western blotting were obtained from EMD Millipore (Schwalbach, Germany). Calebin A (CA), was a generous gift from Sabinsa Corporation (East Windsor, NJ, United States). Calebin A was diluted and 10,000 µM stock solution was prepared in DMSO, and this was further diluted in cell culture medium. Final concentration of DMSO did not exceed 0.1% during the experiments. Focal adhesion kinase inhibitor (FAK-I) (PF-562271) was purchased from Sellekchem (Munich, Germany). Stock sample of 10 mM FAK-I was prepared in DMSO and diluted again in serum-starved medium to make working samples.

### Cell Lines and Cell Culture Conditions

Two distinct colorectal cancer cell lines (HCT116 and RKO), and a stromal fibroblast cell line (MRC-5) were purchased from the European Collection of Cell Cultures (Salisbury, United Kingdom) and a human T-lymphocyte cell line (Jurkat) was obtained from the Leibniz Institute (DSMZ-German Collection of Microorganisms and Cell Cultures). Jurkat was cultured in suspension and HCT116, RKO, and MRC-5 were cultured as monolayers in normal cell growth medium containing 10% FCS under standard conditions (37°C, 5% CO_2_) (Shakibaei et al., 2015).

### Study Design

TME cultures were set up by co-culturing stromal cells (MRC-5), T-lymphocyte cell line (Jurkat) and CRC cells (HCT116, RKO) to investigate the potential of Calebin A on the suppression of epithelial-to-mesenchymal transition in CRC cells as described previously (Buhrmann et al., 2020a). To establish the multicellular TME, CRC cells (HCT116, RKO) in 3-dimensional (3D) alginate cultures (1×10^6^/ml alginate) were transferred to petri dishes containing monolayer fibroblasts (3,000/cm^2^) and in suspension T-lymphocytes (20.000/ml medium). These cells were cultured in serum-starved medium (3% FCS) for 10 days. The CRC cells cultured in alginate beads alone were termed as “basal control”. All experiments were performed with serum starved medium containing only 3% FCS.

### Alginate Culture

As described in detail in our previous studies (Shakibaei and De Souza, 1997; Shakibaei et al., 2015), CRC cells (1x10^6^/ml) were suspended in alginate and dropwise (size: 0.5 cm) added into a sterile CaCl_2_ solution (100 mM) where the drops polymerized into beads after 10 min. After continued washing with NaCl (15 mM), alginate beads were incubated for 1 h in serum starved medium. For the experiments, cells were left untreated or treated with CD in a dose dependent manner (0.1, 1, 2, and 4 µg/ml) for 30 min and then encapsulated in alginate beads, and TME cultures were established as described above. Further, primarily untreated TME cultures were treated with Calebin A (1, 2, 5, and 10 µM) or with FAK-I (0.01, 0.1, 1, and 10 µM). As basal control, CRC were encapsulated in alginate beads without fibroblasts or Jurkat and either left untreated or treated with 5 µM Calebin A. Medium was changed three times a week and cultures were investigated after 10 days.

### Viability Assay

Impact of Calebin A on the proliferation of CRC cells in TME cultures was examined by the MTT reagent, as described before (Buhrmann et al., 2020a). Shortly, CRC cells treated as described above were detached from alginate using a sterile sodium citrate solution (55 mM), were re-suspended in altered medium (no phenol red, no vitamin C, 3% FCS) and 10 μl MTT solvent (5 mg/ml) was spread per well in a 96-well plate in triplicate. Incubation was stopped after 2 h by adding 10% Triton x-100/acidic isopropanol and metabolically active cells were estimated using a multiscanner plate ELISA reader (Bio-Rad Laboratories Inc. Munich, Germany).

### Invasion Test

The cell invasion assay was examined using alginate beads (Shakibaei et al., 2015; Buhrmann et al., 2020a). Shortly, 1 x10^6^/ml cells were embedded in alginate and treated as described above. After incubation for 10 days, the noninvasive cells and alginate matrix were removed from the petri dishes while the invasive cells that migrated across the alginate beads adhered and made colonies in opposite to fibroblasts cells on the bottom of the petri dish. These invasive cells were stained with toluidine blue for 1 h and accurate washed two times with PBS and photographed under the light microscope (Zeiss, Germany). The amount of migrating and positively stained attached colonies was quantified and analyzed by counting all colonies.

### Immunofluorescence

Immunofluorescence labeling of Slug was performed in a modified TME as described (Buhrmann et al., 2020a). Shortly, HCT116, and RKO cells were seeded on glass plates (5,000/plate) and after adherence, they were either left untreated or treated with CD (1, 2 µg/ml) for 30 min and then placed on a steel-net bridge in petri dishes containing monolayer of fibroblasts (10.000/cm^2^) and T-lymphocytes in suspension (20.000/ml) to establish TME. Additionally, cultures were either left untreated or treated with Calebin A (1, 5 µM) for 24 h. For basal control, CRC cells were cultured only on glass plates without creating the TME and either left untreated or were treated with Calebin A (5 µM).

### Western Blot Analysis

Samples from the TME were processed in the same manner as earlier detailed (Shakibaei et al., 2015; Buhrmann et al., 2017). Shortly, alginate samples were suspended in sterile sodium citrate solution for 20 min, lyzed on ice (50 mM Tris/HCl, pH 7.2/150 mM NaCl/(v/v) Triton X-100/1 mM sodium orthovanadate/50 mM sodium pyrophosphate/100 mM sodium fluoride/4 µg/ml pepstatin A/1 mM PMSF), total protein content was measured, and fractionated by SDS-PAGE under reducing conditions. After blotting on a nitrocellulose membrane (Transblot apparatus, Bio-Rad, Munich, Germany), membranes were incubated overnight with primary antibody (1:10,000) at 4°C and incubated for 1.5 h with secondary antibody (1:10,000). Positive binding was determined using nitroblue tetrazolium and 5-bromo-4-chloro-3-indoyl phosphate (VWR, Germany), quantification of bands was performed using the “Quantity One” program (Bio-Rad, Munich, Germany).

### DNA-Binding Assay

To address the role of Calebin A in modulating NF-κB attachment on DNA, we performed a DNA-binding assay with dithiothreitol (DTT) as earlier detailed (Buhrmann et al., 2020a). TME cultures were established and then nuclei and cytoplasm were separated from each other, from CRC cells that remained untreated and/or treated with Calebin A (1, 2, and 5 µM) plus DTT (5 mM) alone or in both for 1 h, as has been thoroughly outlined in previous work (Buhrmann et al., 2020a; Buhrmann et al., 2020b; Buhrmann et al., 2020c). The nuclei were then prepared, separated by SDS-PAGE and blotted as indicated earlier. PARP was used to normalize the samples to the controls.

### Immunoprecipitation Assay

The immunoprecipitation assay was performed as outlined (Shakibaei et al., 2012) to explore the endogenous interaction of NF-κB and Slug on CRC grown in TME cultures. Whole cell extracts of HCT116 and RKO from TME cultures were incubated with 25 µl of either normal rabbit IgG-serum or normal mouse IgG-serum and *Staphylococcus aureus* cells as pre-clearing. Further, samples were incubated with primary antibodies (anti-p-NF-κB-p65 or anti-Slug) diluted in washing buffer [0.1% Tween 20, 150 mM NaCl, 50 mM Tris-HCl (pH 7.2), 1 mM CaCl_2_, 1 mM MgCl_2_ and 1 mM PMSF] for 1.5 h (4°C), with *S. aureus* cells for 1 h and washed three times with washing buffer and once with 50 mM Tris-HCl (pH 7.2). Finally, samples were boiled in SDS-PAGE sample buffer and immunoblotting (anti-p-NF-κB-p65 or anti-Slug) was performed as described above. Additionally, samples were incubated with non-immune rabbit anti-mouse IgG alone as control immunoprecipitations.

### Statistics

Each individual assay was tested separately three times using three separate sets of replicates. A Wilcoxon-Mann-Whitney test was used for statistical analysis. Results were presented as mean ± SD or SEM and compared by one-way, two-way or three-way ANOVA using SPSS Statistics if the normality test was satisfied (Kolmogorov-Smirnov test). A *p*-value of <0.05 was regarded as evidence of statistically important results.

## Results

**FIGURE 1:**
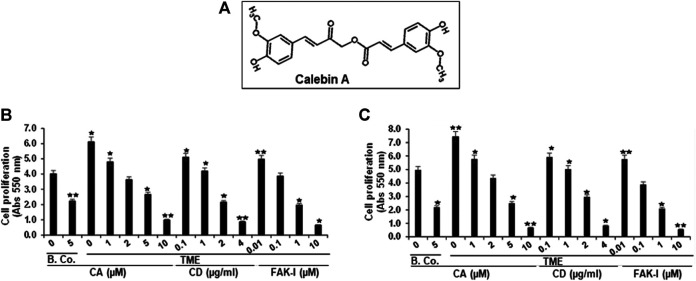
Calebin A or FAK inhibitor or cytochalasin D action on colorectal cancer cell survival. **(A)** Chemical bodies of Calebin A. **(B)** HCT116 and **(C)** RKO cells in alginate beads by themselves (basal control = B. Co.) or in TME were firstly not treated, and secondly, were exposed to a series of doses of Calebin A (CA) (1, 2, 5, 10 μM) or cytochalasin D (CD) (0.1, 1, 2, and 4 µg/ml) or FAK inhibitor (FAK-I) (0.01, 0.1, 1, and 10 µM) for 10 days and cell survival was monitored by MTT. All tests were checked on three different occasions each. *p < 0.05, **p < 0.01 relative to basal control.

The objective of this presentation was to investigate the sub-regulatory actions of Calebin A (a component of *C. longa*) (Figure 1A) on CRC cells in a pro-inflammatory multicellular TME model to represent an *in vivo* approach TME. In this work, we show for the first time that Calebin A suppresses TME-induced CRC cell invasion and malignancy by modulating EMT signaling during the early onset of CRC.

### Calebin A Blocks TME-Induced Viability and Proliferation of CRC Cells, Comparable to FAK Inhibitor or Cytochalasin D

Given that the organization and rearrangement of the cytoskeleton is essential for the cancer cell invasion, migration, and proliferation (Tai et al., 2015), we first investigated whether Calebin A modifies colorectal cancer cell proliferation and viability in TME, similar to FAK inhibitor or cytochalasin D. Serum-starved HCT116 or RKO cells were kept untreated or treated with different concentrations of Calebin A or FAK inhibitor or CD. Cell viability and proliferation were determined using MTT assay as outlined in Materials and Methods. TME significantly increased cell viability and proliferation of HCT116 and RKO cells compared with basal control (Figures 1B,C). In contrast, Calebin A, similar to FAK inhibitor, or CD reduced markedly TME-promoted cell viability and proliferation of cells in 3D-alginate tumor cultures in both basal control and TME in a dose-dependent way. Collectively, the anti-proliferative action of Calebin A resembles FAK inhibitor or cytochalasin D and these proteins may play a role in its efficacy.

### Calebin A Blocks TME-Induced 3D-Alginate CRC Cell Migration and Invasion, Comparable to Cytochalasin D

**FIGURE 2:**
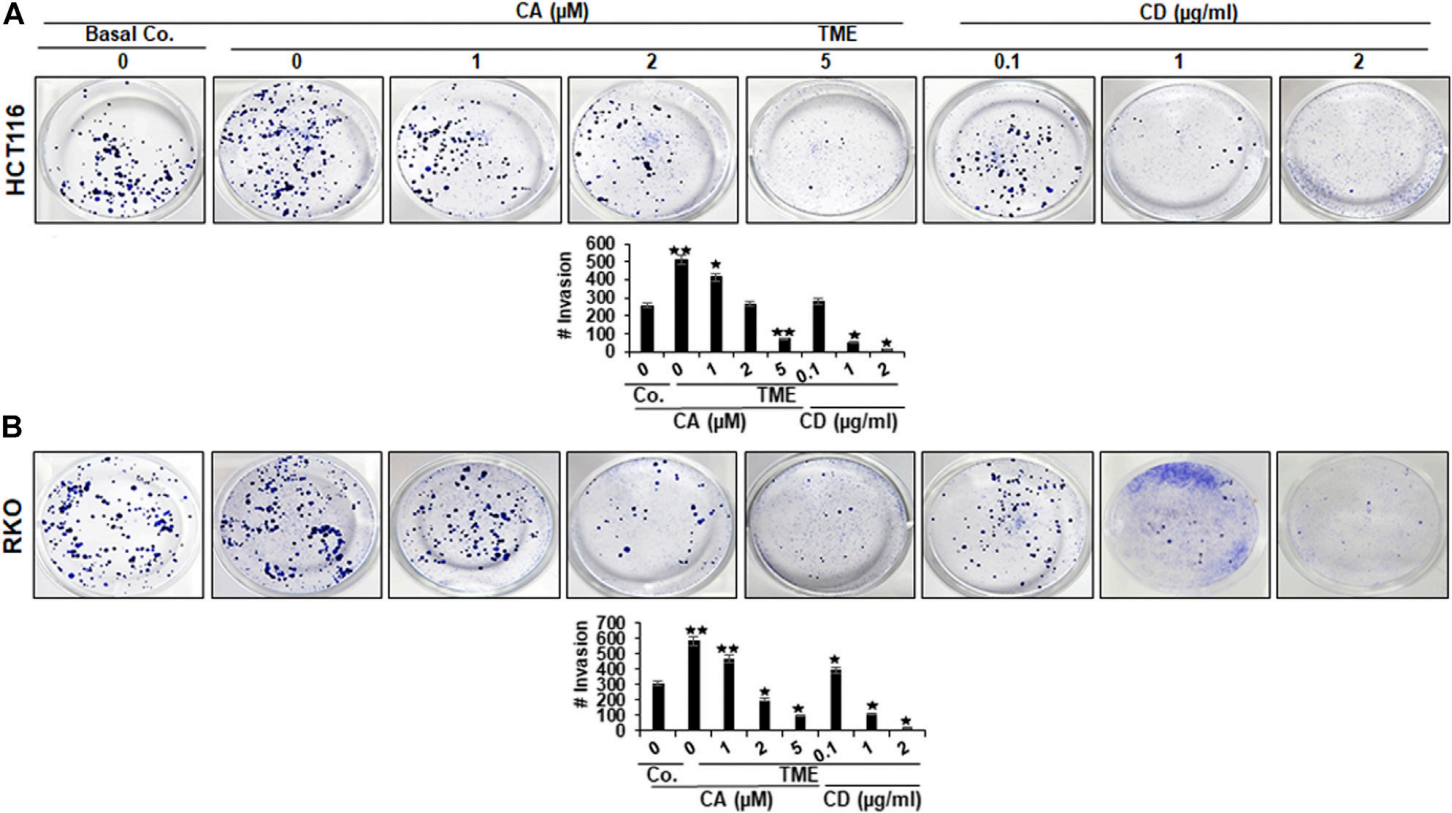
Calebin A or cytochalasin D actions on colorectal cancer cells migration in TME. **(A)** HCT116 and **(B)** RKO cells in alginate beads by themselves (Basal Co.) or in TME were treated with increasing concentration of Calebin A (CA) (1, 2, and 5 μM), of cytochalasin D (CD) (0.1, 1, and 2 µg/ml) or culture medium as control for 10 days in alginate and migrated spheroids were identified by toluidine blue-based labeling as outlined in the Materials and Methods. Results of all tests were scored at a minimum of three counts. *p < 0.05, **p < 0.01 relatively to basal control.

To assess the action of Calebin A or CD on the motility and invasion ability of CRC cells through the TME, HCT116, and RKO cells were grown in a 3D-alginate-matrix TME that more closely represents *in vivo* conditions and handled as outlined in detail in Materials and Methods. As shown in Figures 2A,B, the untreated TME significantly promoted tumor cell migration in CRC cell through the 3D-alginate-based matrix compared with the basal untreated TME control. In contrast, treatment of CRC cells with Calebin A (1, 2, and 5 µM), similar to CD (0,1, 1, and 2 µg/ml), blocked CRC cell migration and invasion across the alginate matrix in TME cultures in a dose-dependent way. Taken together, this is consistent with the results of the MTT assay, which showed that Calebin A, similar to CD inhibited CRC cell viability and proliferation, and these data highlight the importance of the involvement of cytoskeletal signaling proteins in the anti-metastasis properties supported by Calebin A in TME.

### Calebin A Down-Regulated EMT-Related Transcription Factor (Slug) in CRC Cells Comparable to Cytochalasin D, as Shown by Immunofluorescence Microscopy

**FIGURE 3:**
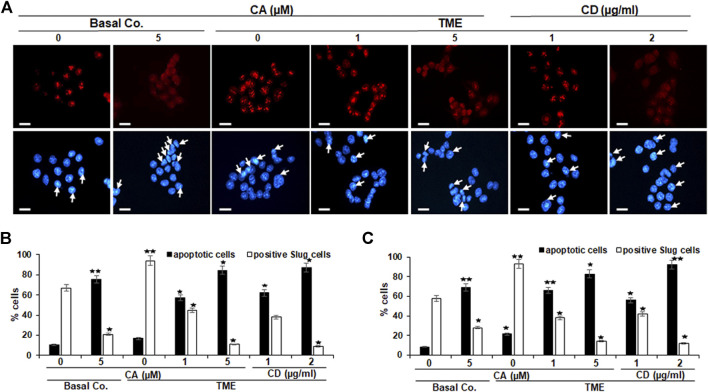
Calebin A or cytochalasin D action on EMT-related transcription factor (Slug) expression in colorectal cancer cells. HCT116 **(A–B)** and RKO **(C)** cells by themselves (basal co.) subjected to Calebin A (CA) (5 µM), or in TME were kept alone untreated, subjected to graded doses of Calebin A (CA) (1, 5 μM) or cytochalasin D (CD) (1, 2 µg/ml) for 4 h in monolayer cultures and underwent immunofluorescence staining with anti-Slug and nuclear counterstaining with DAPI as stated in the Materials and Methods of this study. Magnification 400x; bars = 30 nm. All assays were performed three times each, and the number of apoptotic nuclei (arrows) has been determined by scoring 400–500 cells taken at 25 separate microscopic areas **(B–C)**. *p < 0.05, **p < 0.01 compared to basal control.

Slug is a component of the Snail generation of zinc-finger transcription factors (Blanco et al., 2002), and it has been proposed that Slug is participating in the modulation of cell motility during tumor cell invasion and migration (Cobaleda et al., 2007). To explore the impact of Calebin A on Slug expression in CRC cells, HCT116 and RKO cultures were left untreated or treated with different concentrations of Calebin A or CD and immunolabeled with anti-Slug as described above and evaluated by immunofluorescence microscopy and nuclear labeling with DAPI. In the control TME cultures, HCT116 and RKO cells showed significantly stronger labeling for Slug in the nucleus compared with the basal control (Figures 3A–C). Calebin A treatment, similarly to CD, significantly downregulated the expression of Slug in CRC cells and its nuclear localization in TME cultures, indicating the important pro-inflammatory effects of TME on CRC cells that support tumor promotion. Taken together, these results highlight that Slug is one of the major target proteins of Calebin A for its anti-metastatic or anti-EMT mechanisms in CRC cell TME (Figures 3A–C). Apoptosis was in fact verified by analyzing the apoptosis indicating morphological alterations in the nucleus with DAPI nuclear labeling (Figures 3A–C). Nuclear staining showed that untreated control HCT116 (Figures 3A,B) and RKO (Figure 3C) cell cultures had generally normal nuclear dimensions with only a few apoptotic nuclei in the cultured cells. In contrary, a significant increase in apoptotic cells was observed in cells treated with Calebin A or CD in HCT116 or RKO cells with nuclear morphological alterations (pyknosis, fragmented nuclei, chromatin condensation) in a dose-dependent fashion (Figures 3A–C). Taken together, these outcomes are in agreement with MTT findings and indicate Calebin A has the ability to act to suppress the pro-tumor actions of TME in CRC cells through repression of EMT as well as induction of apoptosis.

### Calebin A Suppressed TME-Induced p-NF-κB-p65, FAK, and Activated TME-Inhibited Caspase-3, Comparable to Cytochalasin D, in CRC Cells

In the past, TMEs have been shown to promote NF-κB activation and phosphorylation, which has been linked to tumor cell migration and metastasis (Ghosh et al., 1998; Lind et al., 2001; Cho et al., 2007; Sutnar et al., 2007). Therefore, we investigated the expression and phosphorylation of NF-κB in relation to malignancy and metastasis of CRC cells. Starved HCT116 and RKO cells were grown in alginate matrix beads and co-cultured in TME conditions, followed by treatment with incremental concentrations of Calebin A (1, 2, and 5 μM), CD (0.1, 1, and 2 µg/ml) or in culture medium as a control for 10 days as detailed in the Materials and Methods. Samples were immunolabeled using antibodies to p65, phospho-specific p65-NF-κB, p-FAK, and Caspase-3.

**FIGURE 4:**
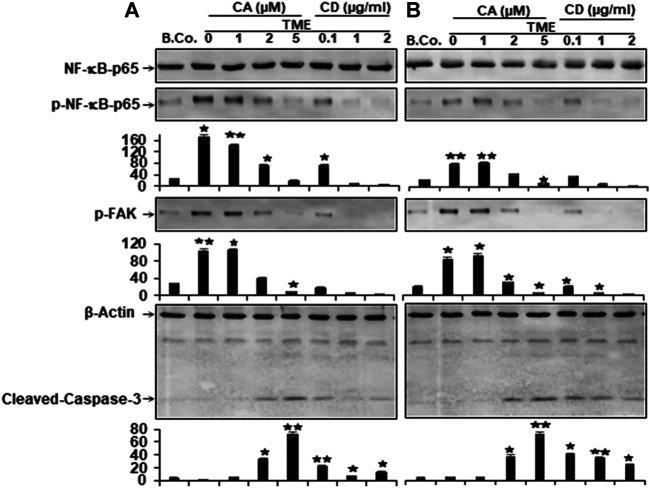
Calebin A or cytochalasin D actions on NF-κB, FAK, and caspase-3 expression in colorectal cancer cells. **(A)** HCT116 and **(B)** RKO cells in alginate beads (B. Co.) or in TME were kept by themselves or treated with variable amounts of Calebin A (CA) (1, 2, and 5 μM) or cytochalasin D (CD) (0.1, 1, and 2 µg/ml) for 10 days as outlined in Materials and Methods. Immunoblotting on total samples was performed through Western blotting using antibodies to p65-NF-κB, phospho-p65-NF-κB, p-FAK, and Caspase-3. Indicated are the results of at least three separate assays, with β-Actin serving as control. The densitometric analysis has been carried out for the indicated proteins. *p < 0.05, **p < 0.01 relatively to basal control.

The levels of p65-NF-κB and p-FAK phosphorylation of HCT116 and RKO-alginate cultures were found to be notably increased in TME compared with basal control cultures. Interestingly, in a manner consistent with the disruption of the cytoskeleton by CD, treatment of TME with Calebin A markedly down-regulated the phosphorylation of p-NF-κB-p65 and p-FAK in a concentration-dependent way in both cells (Figure 4). Calebin A based treatment of TME, similar to CD disruption of the cytoskeleton, significantly upregulated cleaved Caspase-3 protein expression in CRC cells in a dose-dependent way (Figure 4). Contrary, treatment with CD resulted in less cleaved Caspase-3 protein compared with Calebin A, and cleavage of Caspase-3 was greater in RKO than in HCT116 CRC cells (Figure 4). These results suggest that Calebin A suppresses TME-mediated migration and invasion in CRC cells *via* inhibition of NF-κΒ, p-FAK and stimulation of caspase-3.

### Calebin A Modulated TME-Induced TGF-β1 Signaling, Comparable to Cytochalasin D in CRC Cells

**FIGURE 5:**
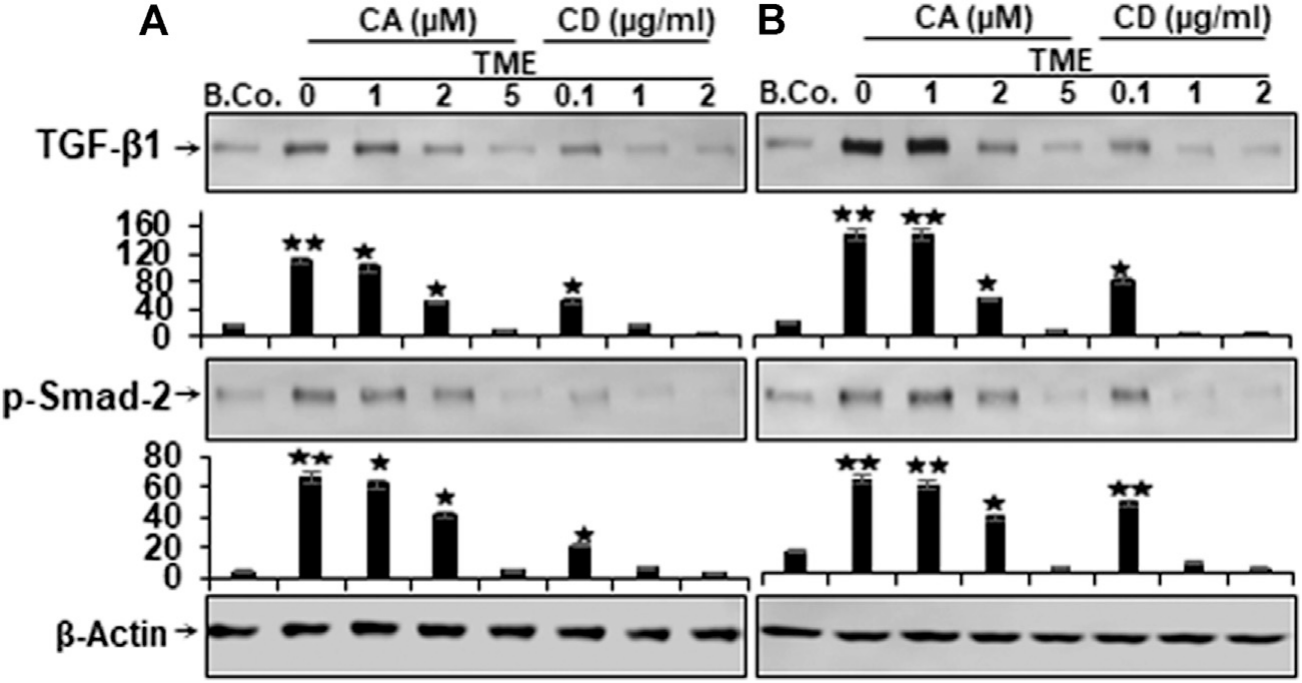
Calebin A or cytochalasin D actions on TGF-β1 and p-Smad-2 expression in colorectal cancer cells. **(A)** HCT116 and **(B)** RKO cells in alginate beads (B. Co.) or in TME were maintained untreated or treated as outlined to the previous. Immunoblotting of total samples was performed by Western blotting using antibodies to TGF-β1 and p-Smad-2. Indicated are the results of at least three separate assays, with β-Actin serving as loading control. Densitometric analysis was performed for the indicated proteins. *p < 0.05, **p < 0.01 relatively to basal control.

The cytokine transforming growth factor-β1 (TGF-β1) is a crucial factor in tumor progression and a strong enhancer of EMT during tumor invasion and metastasis (Massagué, 2000; Siegel and Massagué, 2003; Derynck and Akhurst, 2007), therefore, next, to determine whether TGF-β1 is implicated in augmenting tumor cell proliferation, migration, and the factors that stimulate cancer, we studied TGF-β1 protein expression in HCT116 and RKO cells. Starved cells grown in the TME cultures were either kept untreated or treated as mentioned in the “Materials and Methods” paragraph. Both CRC cells exhibited low expression of TGF-β1 and p-Smad-2 proteins in basal control (Figure 5). Whereas, immunoblotting showed substantially increased levels of TGF-β1 and p-Smad-2 expression in both HCT116 and RKO cells obtained by TME (Figure 5). Importantly, in a manner resembling CD-treated cultures, Calebin A was found to considerably down-regulate the expression of the biomarkers mentioned above in a concentration-dependent way (Figure 5). Taken together, these outcomes are consonant with those of several other studies documenting that TGF-β1 and cytokines are the major contributors to progression, invasion, and metastasis within the TME (Derynck and Akhurst, 2007; Li et al., 2014; Jang et al., 2015). Collectively, Calebin A acts against the TMEs and has a potent suppressive effect on the mentioned processes.

### Calebin A Modulated TME-Induced Altering the Expression of EMT-Specific Biomarkers, Comparable to Cytochalasin D in CRC Cells

**FIGURE 6:**
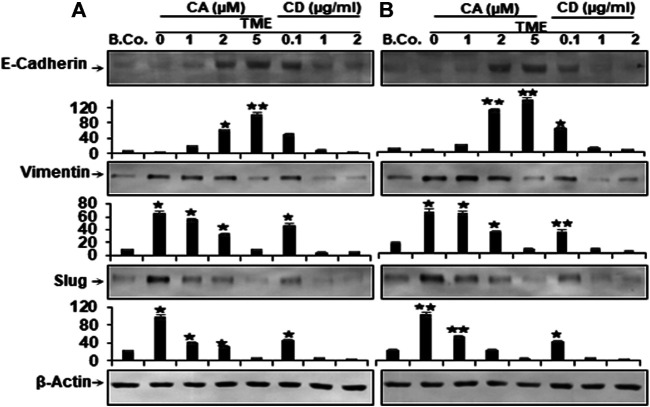
Calebin A or cytochalasin D actions on EMT-specific biomarkers in colorectal cancer cells. HCT116 **(A)** and RKO cells **(B)** in alginate beads (B. Co.) or in TME were maintained alone or treated as outlined to the previous. Immunoblotting of total samples was performed by Western blotting using antibodies to E-Cadherin, vimentin and Slug. Indicated are the results of at least three separate assays, with β-Actin serving as loading control. Densitometric analysis was performed for the indicated proteins. *p < 0.05, **p < 0.01 relatively to basal control.

To learn more about the multifunctional role of the Calebin A, in TME-induced tumor malignancy and metastasis in CRC cells, in relation to motility and EMT, we examined the level of EMT-related pathway protein expression, including E-cadherin, vimentin, and Slug. EMT has been reported to have a major function in carcinogenesis by leading to weakening of cell-cell adhesion, alteration of ECM, and increased motility of cancer cells (Martin, 2014). We investigated the expression of EMT biomarkers for tumorigenicity and the possible effects of Calebin A on these biomarkers. Alginate basal control HCT116 and RKO cells revealed a basal level expression of the aforementioned EMT markers (Figure 6). In contrast, Western blot results showed markedly down-regulated expression of E-cadherin and markedly up-regulated expression of vimentin and Slug in both cells from TME cultures (Figure 6). However, similar to CD, treatment with Calebin A resulted in up-regulation of E-cadherin expression and, in contrast, significant down-regulation of vimentin and Slug expression in a concentration-dependent way, indicating the prominent targeting impact of Calebin A on the EMT signaling pathway (Figure 6). The densitometric analysis of the immunoblots supported the presented findings (Figure 6). Taken together, such data clearly demonstrate the critical function of Calebin A in regulating TME-stimulated EMT pathway, as well as Calebin A mediates anti-metastasis effects in part by down-regulating the EMT pathway of CRC cells in the TME.

### DTT Blocks Calebin A-Suppressive Effects of NF-κB Binding to DNA in CRC Cells

**FIGURE 7:**
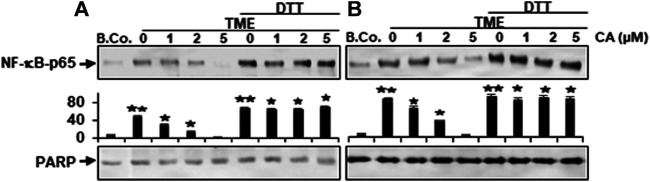
Calebin A acts on TME-induced p-NF-κB-p65 engagement with DNA in colorectal cancer cells. **(A)** HCT116 and **(B)** RKO cells in alginate beads were cultured alone (B. Co.) or in TME were cultured alone or subjected to Calebin A (CA) as outlined in the Materials and Methods. Dissected nuclei of basal control or TME cells that has been allowed to incubate for 30 min with the stated concentrations of Calebin A in the presence or absence of 5 mM DTT were processed, then examined for p-NF-κB-p65 linkage to DNA using Western blotting. PARP acted as control. Densitometric assessment was done for the indicated proteins. *p < 0.05, **p < 0.01 relatively to basal control.

We further wanted to know whether Calebin A has the ability to trigger the repression of NF-κB phosphorylation by directly interfering with its binding to DNA. To address this question, we treated isolated nuclei obtained from HCT116 (A) and RKO (B) cells as indicated in Materials and Methods. To this end, Calebin A markedly attenuated the association of NF-κB binding to DNA in a dose-dependent way, as demonstrated in Figures 7A,B. Since the position 38 cysteine region in p65 protein is required for DNA binding, an assay to remove the cysteine regions was performed with DTT either with or without Calebin A. Indeed, our study revealed that the blocking effect of Calebin A toward the attachment of p65-NF-κB on DNA has been abolished when DTT was applied to the nuclei of RKO cells, similar to HCT116 cells, indicating a specific effect of Calebin A in regulating the interaction of NF-κB with DNA and this is not cell specific (Figures 7A,B). Taken together, these data suggest that Calebin A directly modulates the binding of p65-NF-κB to DNA.

### Calebin A Inhibits TME-Induced p-NF-κB-p65 Association With the Master EMT-Related Transcription Factor (Slug), Comparable to Cytochalasin D in CRC Cells

**FIGURE 8:**
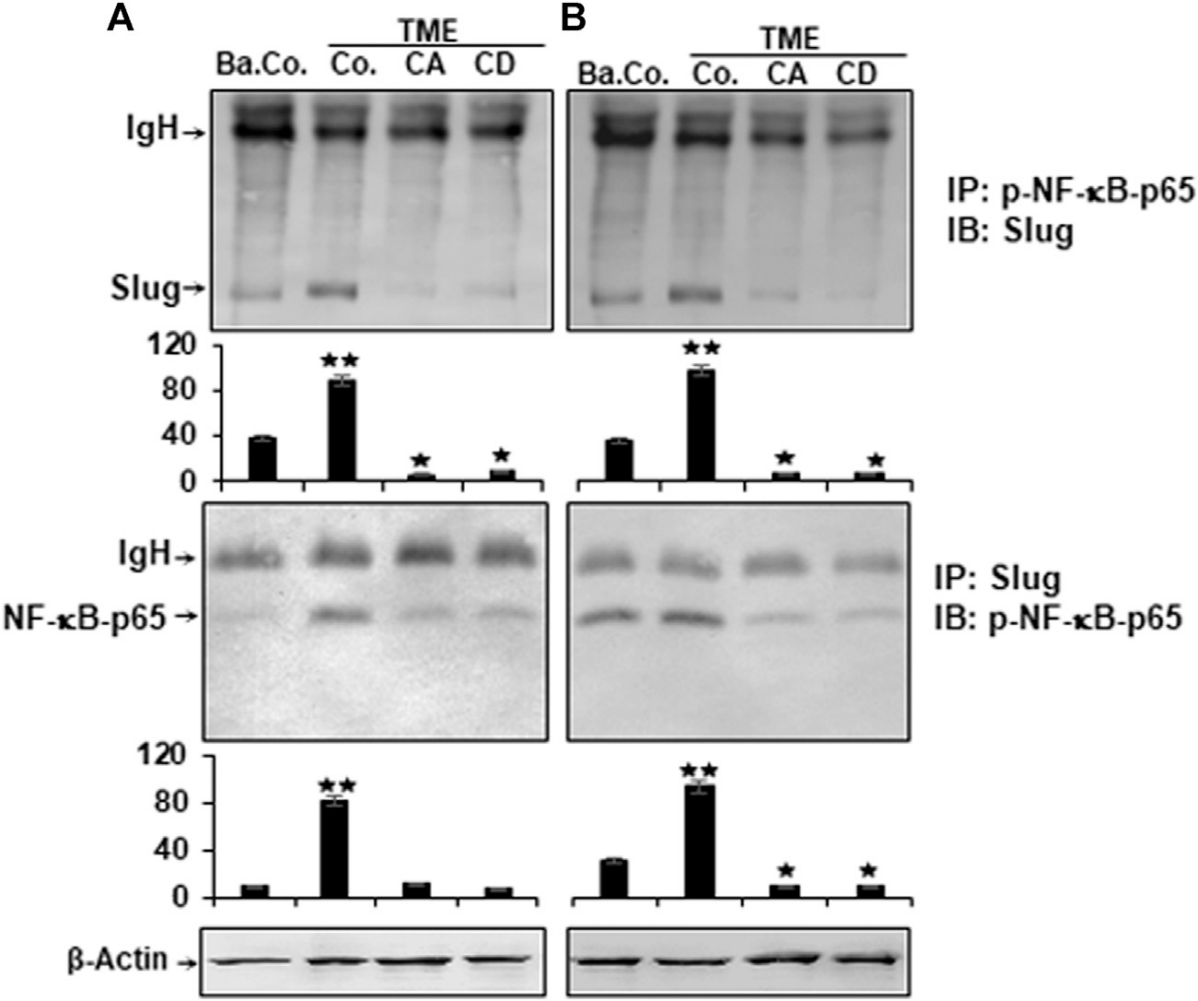
Calebin A or cytochalasin D impact on TME-induced p-NF-κB-p65 association with master EMT-related transcription factor (Slug) in colorectal cancer cells. HCT116 **(A)** and RKO **(B)** cells in alginate matrix alone (B. Co.) or in TME were kept without treatment (Co.) or treated with Calebin A (CA) or Cytochalasin D (CD) as outlined in the Materials and Methods. The lysate samples were immunoprecipitated (IP) using anti-p-NF-κB-p65 or with anti-Slug. Finally, each immunoprecipitates was fractionated by SDS-PAGE and subjected to immunoblotting (IB) with anti-Slug or anti-p-NF-κB-p65. Densitometric assays on protein levels as displayed with immunoblotting were carried out in triplicate. The initial samples were subjected to antibody against β-Actin as control. Values were comparable with the reference control. *p* < 0.05 (*), *p* < 0.01 (**). IgH = immunoglobulin heavy chain.

Cell motility, cytoskeletal remodeling, cell adhesion, FAK signaling pathways, and NF-κB have already been shown to be linked to the regulation of cancer cell growth, viability, and invasiveness (Cary and Guan, 1999; Schlaepfer et al., 1999; Cheng et al., 2001; Aggarwal, 2004; Zhang et al., 2009). The functional link between the proteins that have a metastasis-promoting effect in the TME and their contribution to cancer metastasis is known. We next performed co-immunoprecipitation experiment to provide evidence for a potentially interaction of NF-κB and Slug in CRC cells in TME. Cells were treated as described in Materials and Methods, and cell lysates immunoprecipitated using antibodies directed to p-NF-κB or to Slug, that was subsequently immunoblotted using Slug or p-NF-κB antibodies. As shown in Figure 8, surprisingly, in both CRC cells, we found markedly strong co-immunoprecipitation of NF-κB protein with Slug protein in TME (Figure 8). Marginal co-immunoprecipitation of NF-κB protein with the Slug protein was observed in untreated basal control cultures (Figure 8). However, similar to CD, treatment with Calebin A significantly inhibited this co-immunoprecipitation of NF-κB protein with Slug protein. Densitometric analysis of immunoblots supported the results presented at the top (Figure 8). Overall, our data strongly indicate that, on the one hand TME promotes the formation of the NF-κB/Slug complex during tumorigenesis, resulting in malignancy and metastasis in CRC cells, and, on the other hand Calebin A prevents TME-induced EMT, partly due to blockage of the NF-κB/Slug route.

## Discussion

The present work has allowed us to establish a CRC cell 3D-alginate pro-inflammatory multicellular TME culture mimicking the heterogeneous TME *in vivo* to evaluate the anti-tumor actions observed with Calebin A, mainly the anti-EMT effect. We provided the following insights and investigated the role of TME in promoting CRC tumor progression (tumor migration and EMT) and the modulatory actions of Calebin A in this process. 1) The TME induced viability, proliferation, and alterations in the expression of biomarkers related to EMT (elevated vimentin, depressed E-cadherin), leading to higher motility and invasiveness in CRC cells. 2) However, these effects were dramatically decreased by Calebin A, similar to FAK inhibitor or CD. 3) In addition, TME induced stabilization of master EMT-related transcription factor (Slug), activation of NF-κB, increased TGF-β1- and p-Smad-2-expression, and FAK signaling pathways. 4) Calebin A treatment, similar to CD, significantly reduced TME-induced NF-κB, FAK and Slug signaling and cell migration in both CRC cell lines as well. 5) Calebin A selectively down-regulated the binding of p65-NF-κB on DNA in the two CRC cells, thereby modulating NF-κB-dependent TME-induced EMT signaling. 6) Finally, TME induced a functional connection between NF-κB and Slug, which could enhance Slug-dependent up-regulation of EMT, whereas Calebin A, similar to CD strongly blocked this association of the two transcription factors, thereby blocking EMT.

It has been repeatedly shown in the recent past that three-dimensional tumor cultures have excellent culture conditions compared to two-dimensional tumor cultures, as these cultures have a set of suitable *in vivo* conditions for tumor cell survival (Luca et al., 2013; Shakibaei et al., 2015; Stankevicius et al., 2017). The alginate-based substrate provides the appropriate 3D microenvironment for tumor cells to proliferate, form typical tumor spheroids and, very importantly, provides conditions for tumor cells to migrate through the alginate substrate and eventually metastasize. Interestingly, the alginate bead culture is solubilized readily and the living CRC tumor cells can be tested as to their multiple properties in the early steps of proliferation and metastasis further supporting the TME in a manner comparable to *in vivo* (Shakibaei et al., 2015; Buhrmann et al., 2016).

Increasing evidences indicate that there is a direct strong link between inflammation, inflammatory mediators and tumorigenesis. However, the mechanisms by which TME and inflammation enable tumor progression remain poorly understood. It is now recognized that inflammation-induced migration, EMT and metastasis of tumor cells in the TME is a major target of many clinical challenges in tumor treatment (Cordon-Cardo and Prives, 1999; Coussens and Werb, 2002; López-Novoa and Nieto, 2009). In this report, we have shown in invasion assays that the TME induced invasion of cells and this was significantly down-regulated through Calebin A, similar to CD or FAK-inhibitor. This similar mode of action of Calebin A and CD is due to an important target in the cell, namely cytoskeletal proteins, which are critical and essential for cancer cell proliferation, migration and invasion and thus EMT (Tai et al., 2015). This target of Calebin A was further confirmed by the fact that Calebin A also simultaneously inhibits the enzyme responsible for polymerization of cytoskeletal proteins, FAK. In addition, focal contacts are membrane-bound complex structures that create connection among the cytoskeleton and the ECM. FAK plays an essential role as an integrator of biochemical signals and mechanical stimuli and also dominates in active and motile cells. Furthermore, FAK found to have an integral function in controlling DNA synthesis and cytoskeletal organization, thereby regulating cell division, proliferation, and migration (Zhao et al., 1998; Oktay et al., 1999). However, it has been shown that high expression of FAK and FAK signal transduction pathways in various tumor cells and tumor stem cells results in anti-apoptotic effects and tumor cell survival. Indeed, FAK plays a fundamental role in modulating tumor aggressiveness and metastasis, and high FAK expression is always associated with a negative prognosis of tumors (Hungerford et al., 1996; Almeida et al., 2000; Sonoda et al., 2000; Kong et al., 2015; Tai et al., 2015).

We also demonstrated that the transcription factor NF-κB is one of the important stimulated inflammatory down-stream signaling pathways for TME. Similar to the CD pathway, Calebin A has a specific modulatory effect against TME-promoted activation of NF-κB pathway as well as NF-κB-regulated proteins in CRC cells. This finding and our previous studies are consistent with previous research demonstrating that NF-κB becomes activated in tumor cells in responding to diverse groups of pro-inflammatory substances, including cytokines and TME, and inhibits apoptosis (Baeuerle and Baichwal, 1997; Buhrmann et al., 2019; Buhrmann et al., 2020a). After its translocation into the nucleus, it associates with the targeted sequence in DNA, leading in turn to gene transcription. It has already been reported that NF-κB is a promising target for the development of anti-tumor drugs (Van Antwerp et al., 1996; Wang et al., 1996). Indeed, TME-induced NF-κB, is known to be among the major mediators of cancer progression, and thus proliferation and EMT. Thus, NF-κB is associated with tumor metastasis (Pikarsky et al., 2004; Wu and Zhou, 2010). Therefore, compounds like Calebin A, which can block the phosphorylation of NF-κB, have the ability to block tumor promotion and metastasis.

Our data further showed that cytokine transforming growth factor-β1 (TGF-β1) and simultaneously, p-Smad-2 expression was significantly up-regulated in TME in both CRC cells. Indeed, it has been previously reported that TME-induced TGF-β1 expression in CRC cells in a Smad2-dependent process (Jang et al., 2015), and it is known to play a major role in TGF-β1-induced tumor progression, migration, and EMT (Li et al., 2014). TGF-β1 induces the signaling pathway for EMT *via* a heteromeric complex of two transmembrane serine/threonine kinase receptors type I and type-II. This leads to phosphorylation of Smad2 and 3 and 4, whereupon these trimers are transported to the nucleus, where they associate with transcription factors such as Snail and Slug to repress the expression of epithelial markers and induce the expression of mesenchymal markers at the mRNA level (Zhang et al., 2018). Moreover, TGF-β1 has been shown to promote tumor cell progression and metastasis mainly through EMT and EMT-linked proteins during tumor invasion and metastasis (Buhrmann et al., 2016; Kaowinn et al., 2017).

Interestingly, the concomitant expression of epithelial cell marker (E-cadherin) was markedly decreased and the expression of mesenchymal biomarkers (vimentin and Slug) was markedly increased in the TME. Indeed, down-regulated E-cadherin expression is known to be a specific feature of the EMT process in tumorigenesis (Bendris et al., 2012). Our results clearly show that Calebin A reversed TME-induced changes in the expression of TGF-β1, p-Smad-2, and EMT biomarkers, similar to CD, in a concentration-dependent manner, suggesting that TME-stimulated EMT can be attenuated in CRC cells. Furthermore, available evidence has recently clearly demonstrated that both genetic and epigenetic influences and factors play important roles in silencing E-cadherin expression in various tumor types (Christofori and Semb, 1999; Cheng et al., 2001). Indeed, it has been further reported that there are some inhibitors of E-cadherin such as the Snail family of zinc finger transcription factors. These transcription factors, as well as Slug, have now been shown to be important modulators of EMT and many other important processes *via* the influence on E-cadherin in various tumors (Batlle et al., 2000; Cano et al., 2000; Grooteclaes and Frisch, 2000; Comijn et al., 2001; Perez-Moreno et al., 2001; Blanco et al., 2002). It was further reported and clearly demonstrated that Slug, a major EMT-related transcription factor, has a specific repressive effect on the mouse E-cadherin promoter. Besides, overexpression of Slug in MDCK cells and breast cancer cell lines have been reported to significantly repress endogenous E-cadherin expression, thereby significantly inhibiting cell EMT (invasion and migration) (Hajra et al., 2002; Bolós et al., 2003).

Moreover, our data show that the TME clearly induces the expression of EMT-related transcription factors such as Slug and NF-κB, indicating targeted EMT activation, and they are known to be highly expressed especially in tumor stem cells and invaded tumor cells (Bhat-Nakshatri et al., 2010; Jang et al., 2015), and Calebin A suppressed the TME-induced expression of Slug and NF-κB. Indeed, a large body of literature indicates that TME-induced cytokines and pro-inflammatory signaling may promote tumor cell metastasis and EMT signaling (Virchow, 1881; Coussens and Werb, 2002; de Visser et al., 2006; Medzhitov, 2008; Balkwill, 2009; Buhrmann et al., 2020a).

We demonstrated further that TME strongly augmented CRC cell migration and invasion by triggering the EMT signaling pathway *via* NF-κB-mediated Slug axis stabilization and activation, which plays a critical role in CRC cell metastasis. These results suggest that a synergistic functional interaction of Slug and NF-κB is required for initiation of EMT and migration of tumor cells. However, when the 3D-alginate CRC tumor cells were treated with Calebin A, similar to CD, NF-κΒ/Slug-axis-activation was markedly inhibited, EMT-related protein expression was significantly reversed, and TME-stimulated active migration and invasion were dramatically reduced.

## Conclusion

**FIGURE 9:**
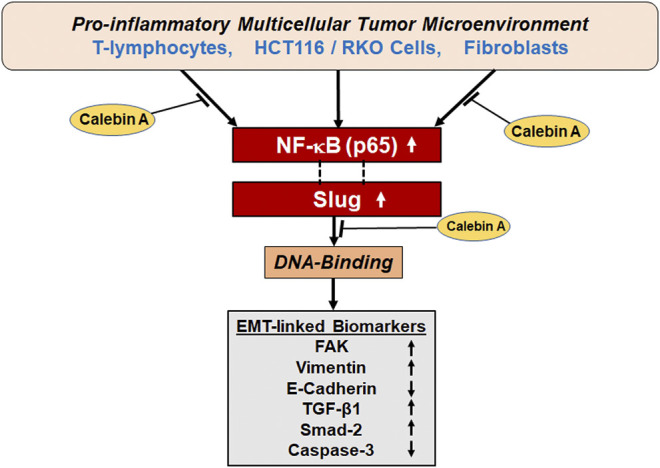
A graphic presentation of the antitumorigenic actions of Calebin A *via* modulation of EMT and Slug/NF-κB axis of colorectal cancer cells in pro-inflammatory multicellular TME.

In conclusion, our results report the first evidence that Calebin A can attenuate the TME-promoted EMT, invasion in CRC cells in 3D-alginate beads, similar to CD or FAK-inhibitor *via* regulation of FAK, EMT biomarkers, and TGF-β1/Smad-2 expression. The inhibitory effect of Calebin A on TME-induced EMT is also associated with suppression of the NF-κB/Slug axis pathway. Thus, as a multitargeted component, Calebin A is a promising therapeutic phytopharmaceutical for the treatment of CRC (Figure 9).

## Data Availability

The raw data supporting the conclusions of this article will be made available by the authors, without undue reservation, to any qualified researcher.
