# Supplementary figures and images for: Evidence That Tumor Microenvironment Initiates Epithelial-To-Mesenchymal Transition and Calebin A can Suppress it in Colorectal Cancer Cells
Source: Front Pharmacol. 2021 Jul 2;12:699842. doi: 10.3389/fphar.2021.699842 (PMC8283792; doi:10.3389/fphar.2021.699842)

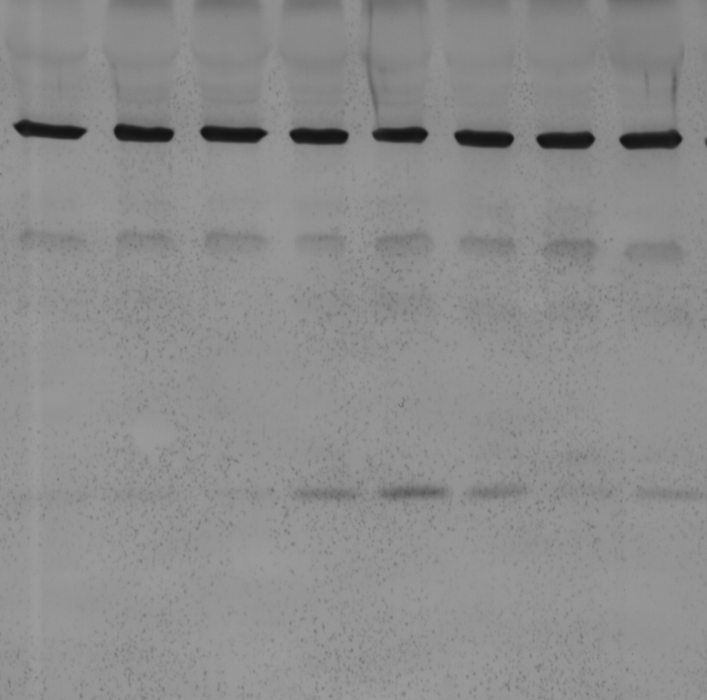

Supplement: Supplementary file 1 [file DataSheet1.ZIP › Buhrmann et al 2021-OriginalFigures-frontiers in Pharmacology/Figure 4/HCT116/HCT116-Caspase-3-ß-Actin-Original.tif]

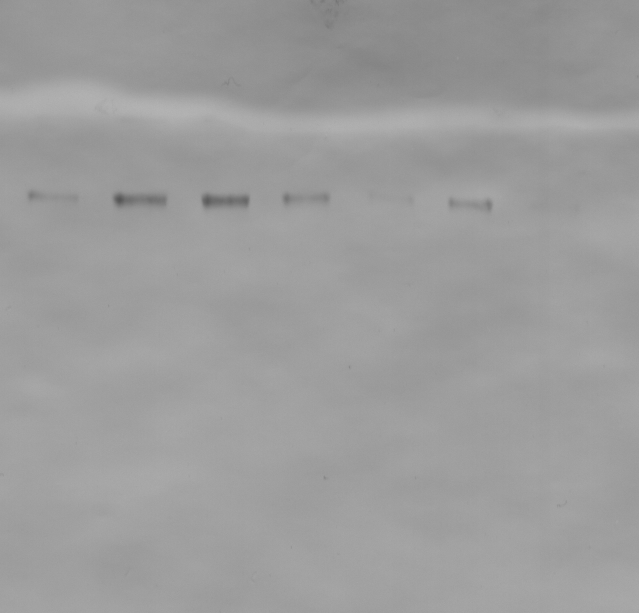

Supplement: Supplementary file 1 [file DataSheet1.ZIP › Buhrmann et al 2021-OriginalFigures-frontiers in Pharmacology/Figure 4/HCT116/HCT116-p-FAK-Original.tif]

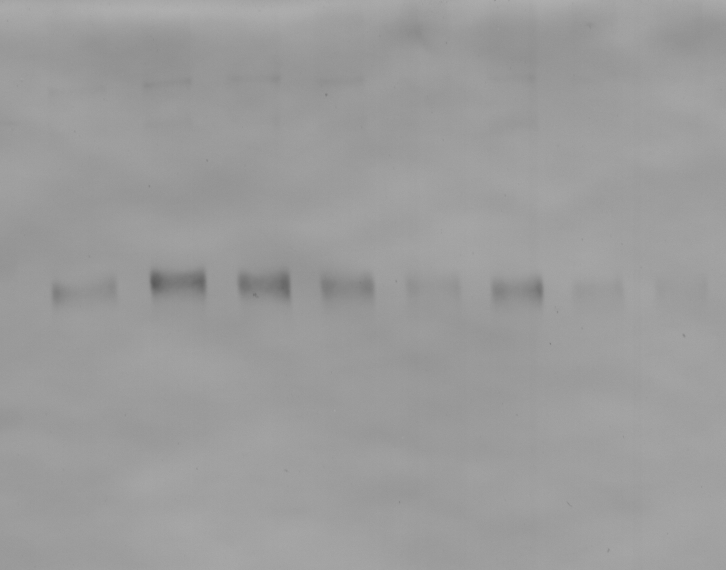

Supplement: Supplementary file 1 [file DataSheet1.ZIP › Buhrmann et al 2021-OriginalFigures-frontiers in Pharmacology/Figure 4/HCT116/HCT116-p-NF-kB-Original.tif]

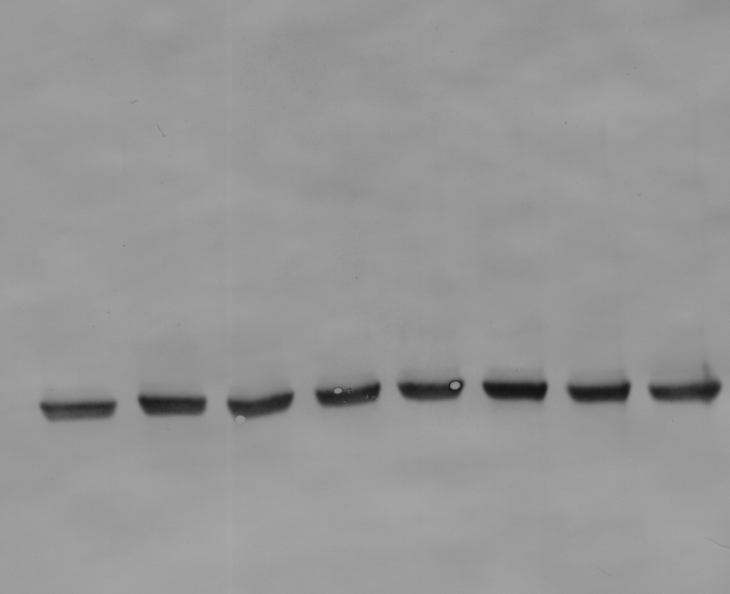

Supplement: Supplementary file 1 [file DataSheet1.ZIP › Buhrmann et al 2021-OriginalFigures-frontiers in Pharmacology/Figure 4/HCT116/HCT116-pan-NFkB-Original.tif]

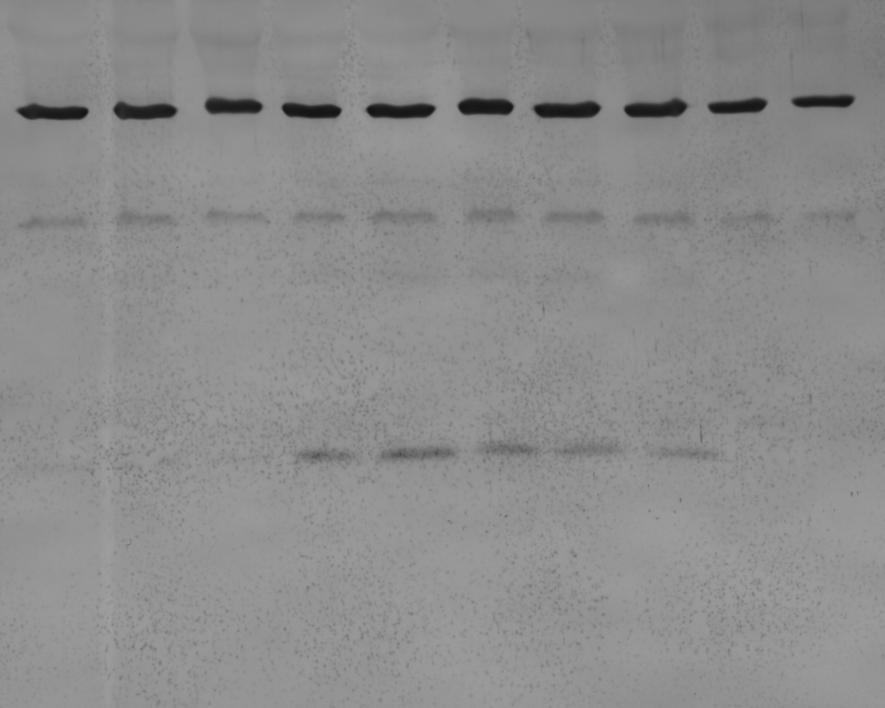

Supplement: Supplementary file 1 [file DataSheet1.ZIP › Buhrmann et al 2021-OriginalFigures-frontiers in Pharmacology/Figure 4/RKO/RKO-Caspase-3-ß-Actin-Original.tif]

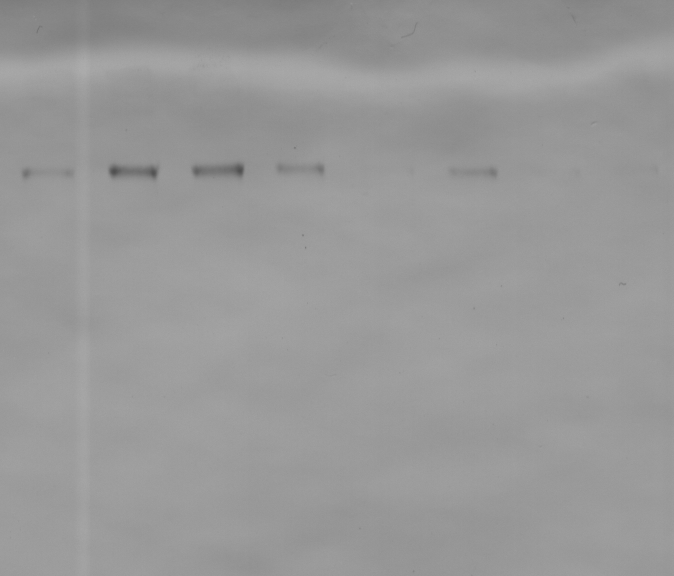

Supplement: Supplementary file 1 [file DataSheet1.ZIP › Buhrmann et al 2021-OriginalFigures-frontiers in Pharmacology/Figure 4/RKO/RKO-p-FAK-Original.tif]

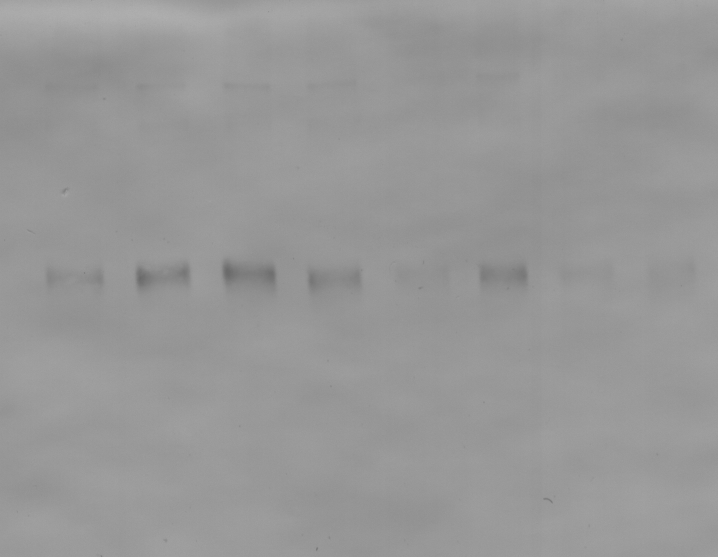

Supplement: Supplementary file 1 [file DataSheet1.ZIP › Buhrmann et al 2021-OriginalFigures-frontiers in Pharmacology/Figure 4/RKO/RKO-p-NF-kB-Original.tif]

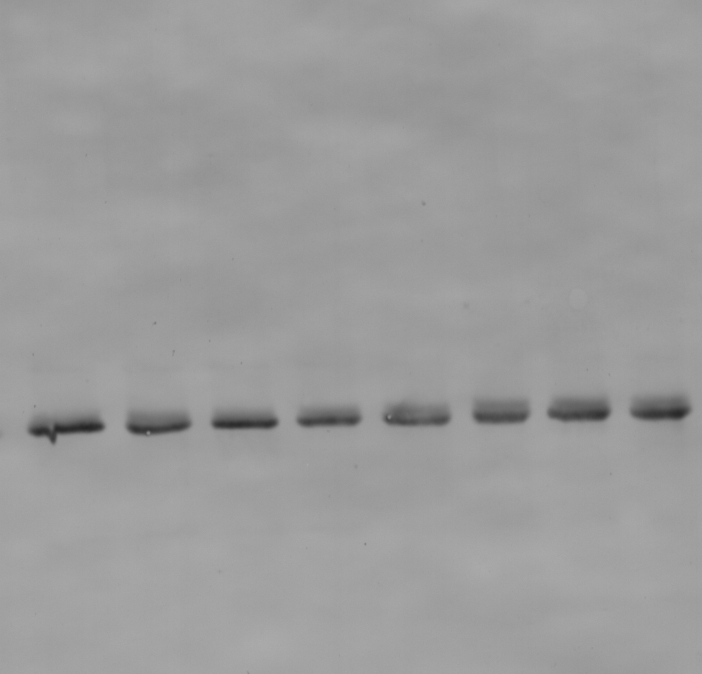

Supplement: Supplementary file 1 [file DataSheet1.ZIP › Buhrmann et al 2021-OriginalFigures-frontiers in Pharmacology/Figure 4/RKO/RKO-pan-NFkB-Original.tif]

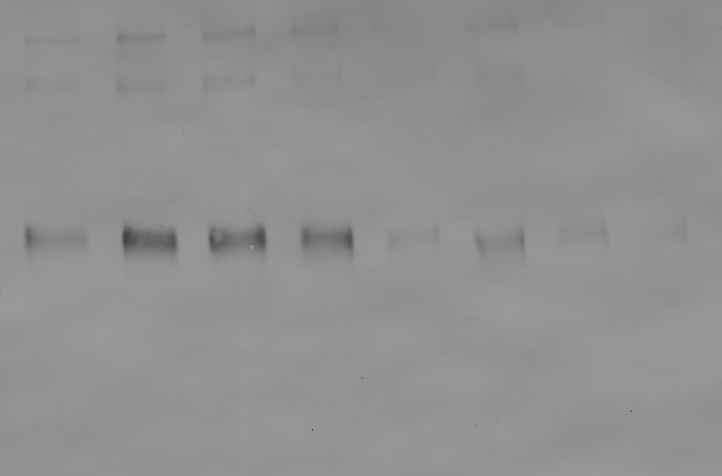

Supplement: Supplementary file 1 [file DataSheet1.ZIP › Buhrmann et al 2021-OriginalFigures-frontiers in Pharmacology/Figure 5/HCT116/HCT116-Smad-2-Original.tif]

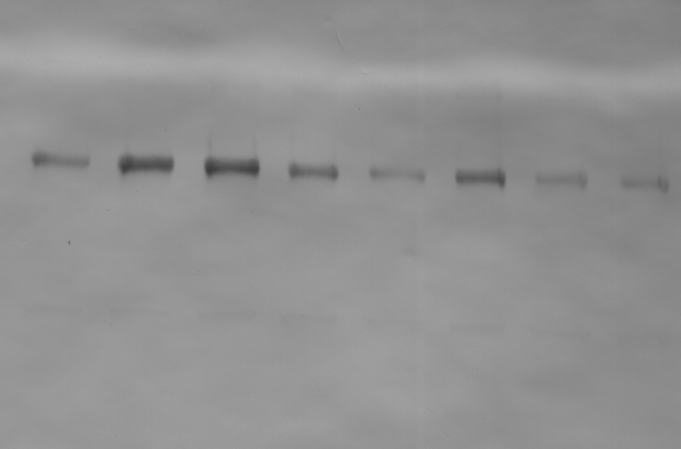

Supplement: Supplementary file 1 [file DataSheet1.ZIP › Buhrmann et al 2021-OriginalFigures-frontiers in Pharmacology/Figure 5/HCT116/HCT116-TGF-ß1-Original.tif]

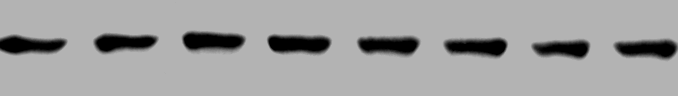

Supplement: Supplementary file 1 [file DataSheet1.ZIP › Buhrmann et al 2021-OriginalFigures-frontiers in Pharmacology/Figure 5/HCT116/HCT116-ß-Actin-Original.tif]

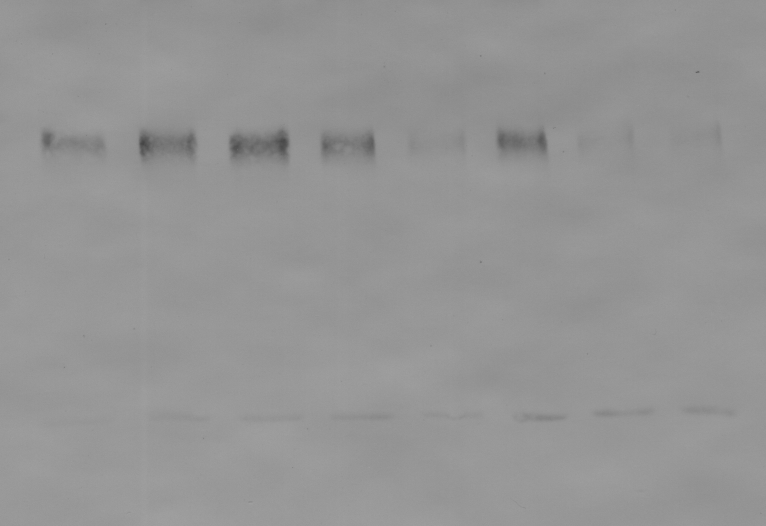

Supplement: Supplementary file 1 [file DataSheet1.ZIP › Buhrmann et al 2021-OriginalFigures-frontiers in Pharmacology/Figure 5/RKO/RKO-p-Smad-2-Original.tif]

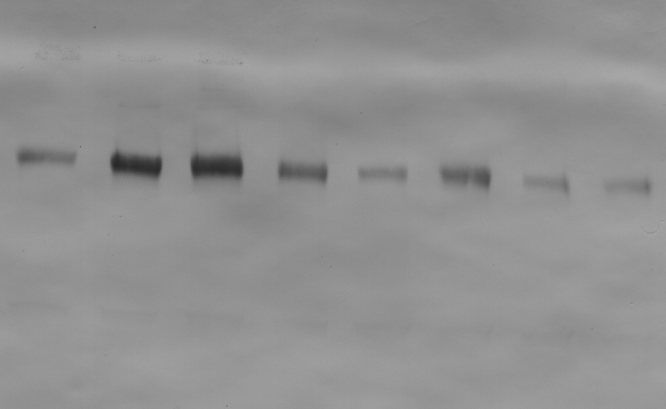

Supplement: Supplementary file 1 [file DataSheet1.ZIP › Buhrmann et al 2021-OriginalFigures-frontiers in Pharmacology/Figure 5/RKO/RKO-TGF-ß1-Original.tif]

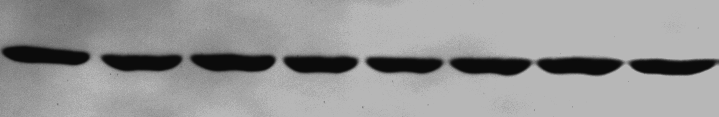

Supplement: Supplementary file 1 [file DataSheet1.ZIP › Buhrmann et al 2021-OriginalFigures-frontiers in Pharmacology/Figure 5/RKO/RKO-ß-Actin-Original.tif]

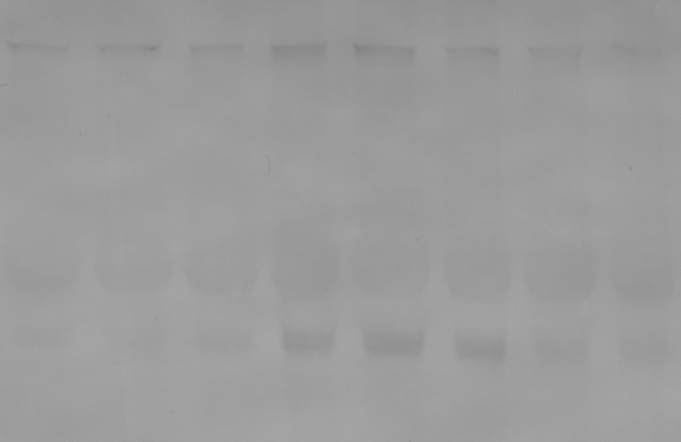

Supplement: Supplementary file 1 [file DataSheet1.ZIP › Buhrmann et al 2021-OriginalFigures-frontiers in Pharmacology/Figure 6/HCT116/HCT116-E-Cadherin-Original.tif]

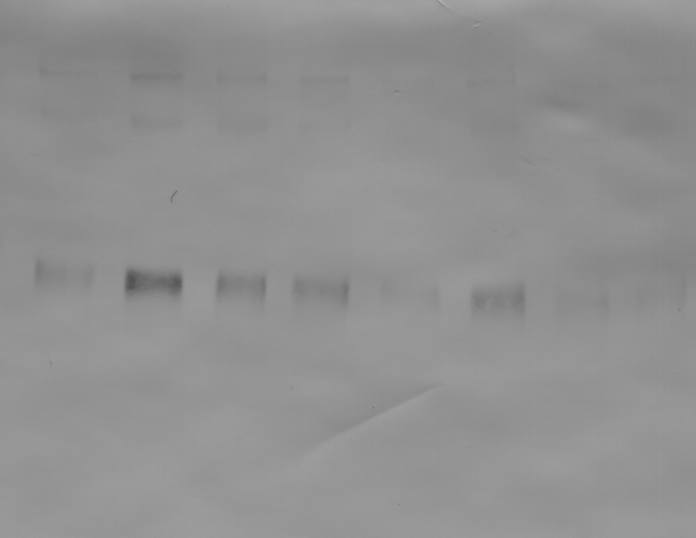

Supplement: Supplementary file 1 [file DataSheet1.ZIP › Buhrmann et al 2021-OriginalFigures-frontiers in Pharmacology/Figure 6/HCT116/HCT116-Slug-Original.tif]

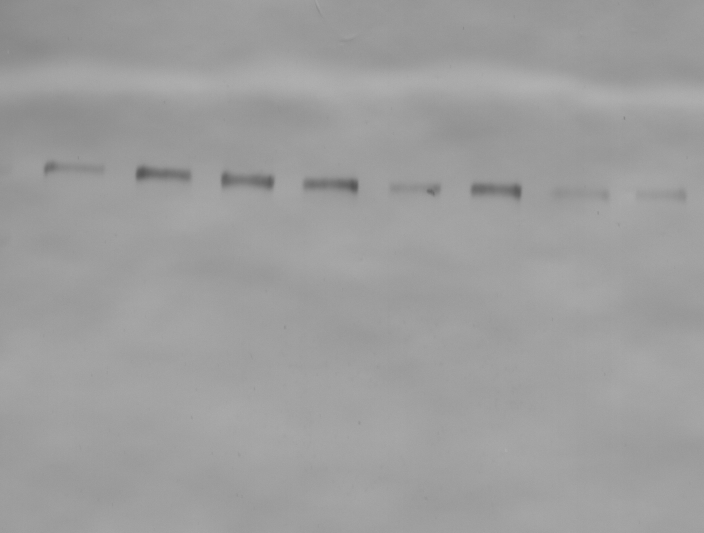

Supplement: Supplementary file 1 [file DataSheet1.ZIP › Buhrmann et al 2021-OriginalFigures-frontiers in Pharmacology/Figure 6/HCT116/HCT116-Vimentin-Original.tif]

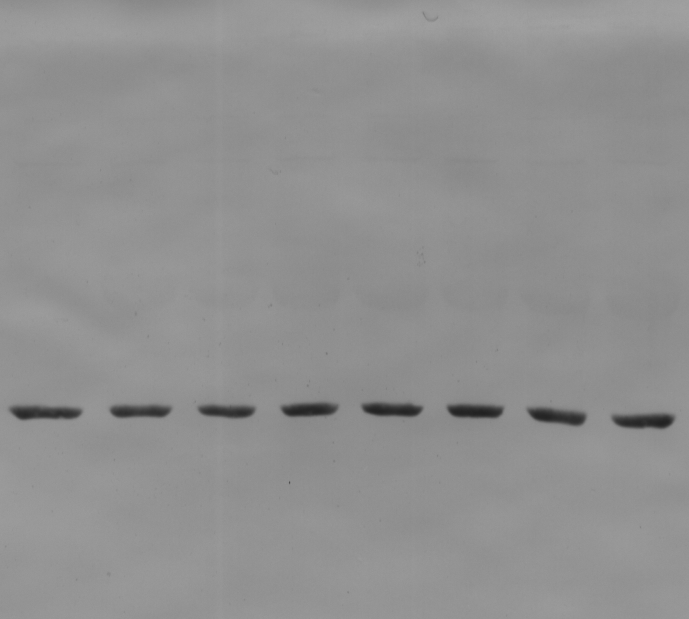

Supplement: Supplementary file 1 [file DataSheet1.ZIP › Buhrmann et al 2021-OriginalFigures-frontiers in Pharmacology/Figure 6/HCT116/HCT116-ß-Actin-Original.tif]

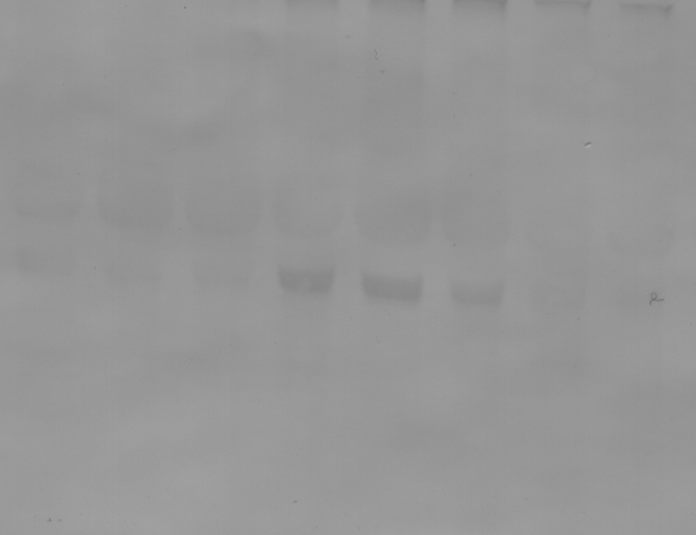

Supplement: Supplementary file 1 [file DataSheet1.ZIP › Buhrmann et al 2021-OriginalFigures-frontiers in Pharmacology/Figure 6/RKO/RKO-E-Cadherin-Original.tif]

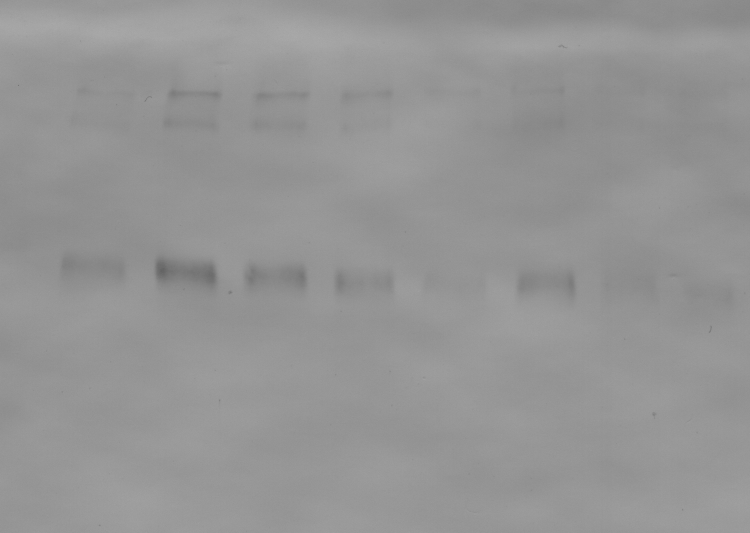

Supplement: Supplementary file 1 [file DataSheet1.ZIP › Buhrmann et al 2021-OriginalFigures-frontiers in Pharmacology/Figure 6/RKO/RKO-Slug-Original.tif]

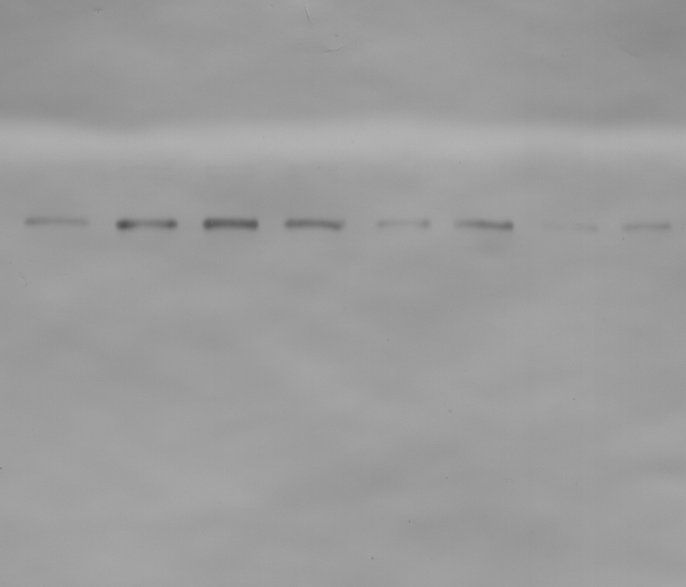

Supplement: Supplementary file 1 [file DataSheet1.ZIP › Buhrmann et al 2021-OriginalFigures-frontiers in Pharmacology/Figure 6/RKO/RKO-Vimentin-Original.tif]

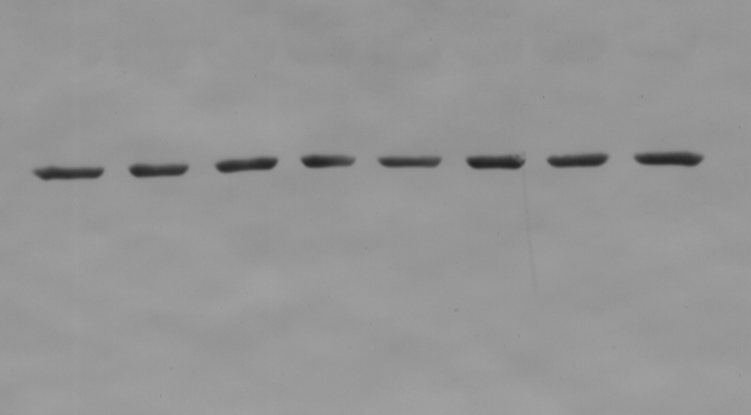

Supplement: Supplementary file 1 [file DataSheet1.ZIP › Buhrmann et al 2021-OriginalFigures-frontiers in Pharmacology/Figure 6/RKO/RKO-ß-Actin-Original.tif]

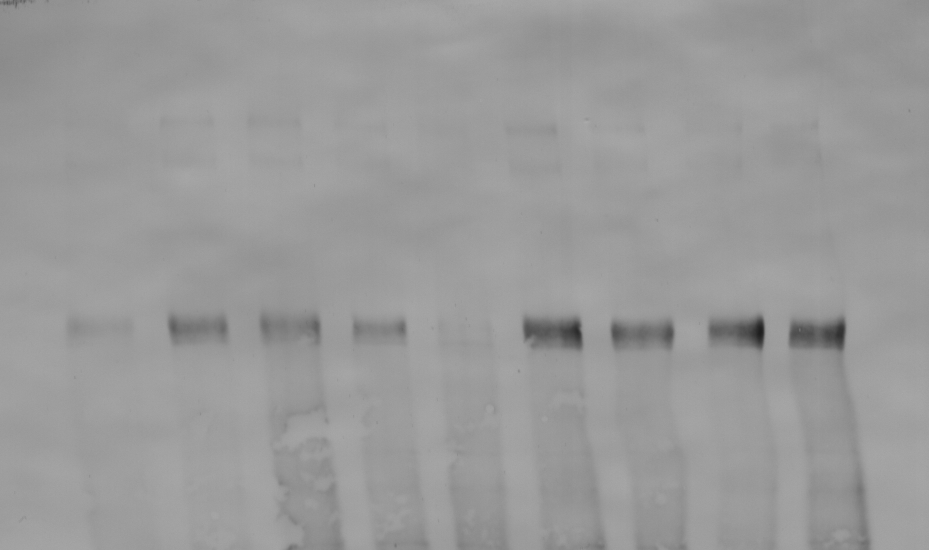

Supplement: Supplementary file 1 [file DataSheet1.ZIP › Buhrmann et al 2021-OriginalFigures-frontiers in Pharmacology/Figure 7/HCT116/HCT116-Bdg-assay-p-NF-kB-Original.tif]

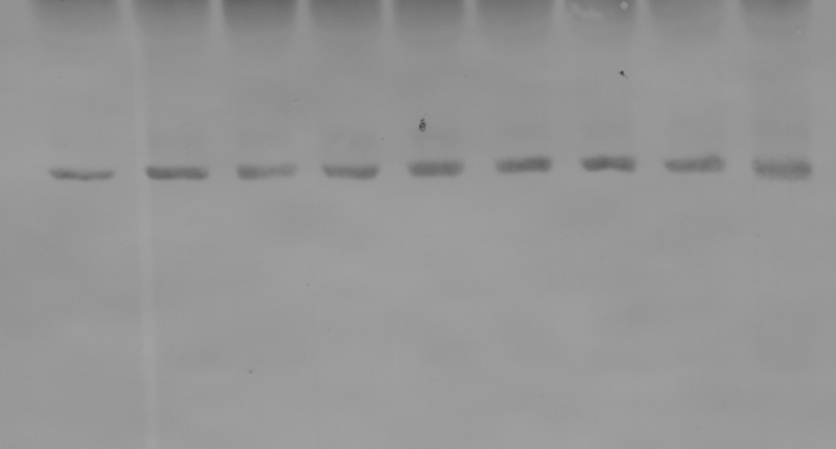

Supplement: Supplementary file 1 [file DataSheet1.ZIP › Buhrmann et al 2021-OriginalFigures-frontiers in Pharmacology/Figure 7/HCT116/HCT116-Bdg-assay-PARP-Original.tif]

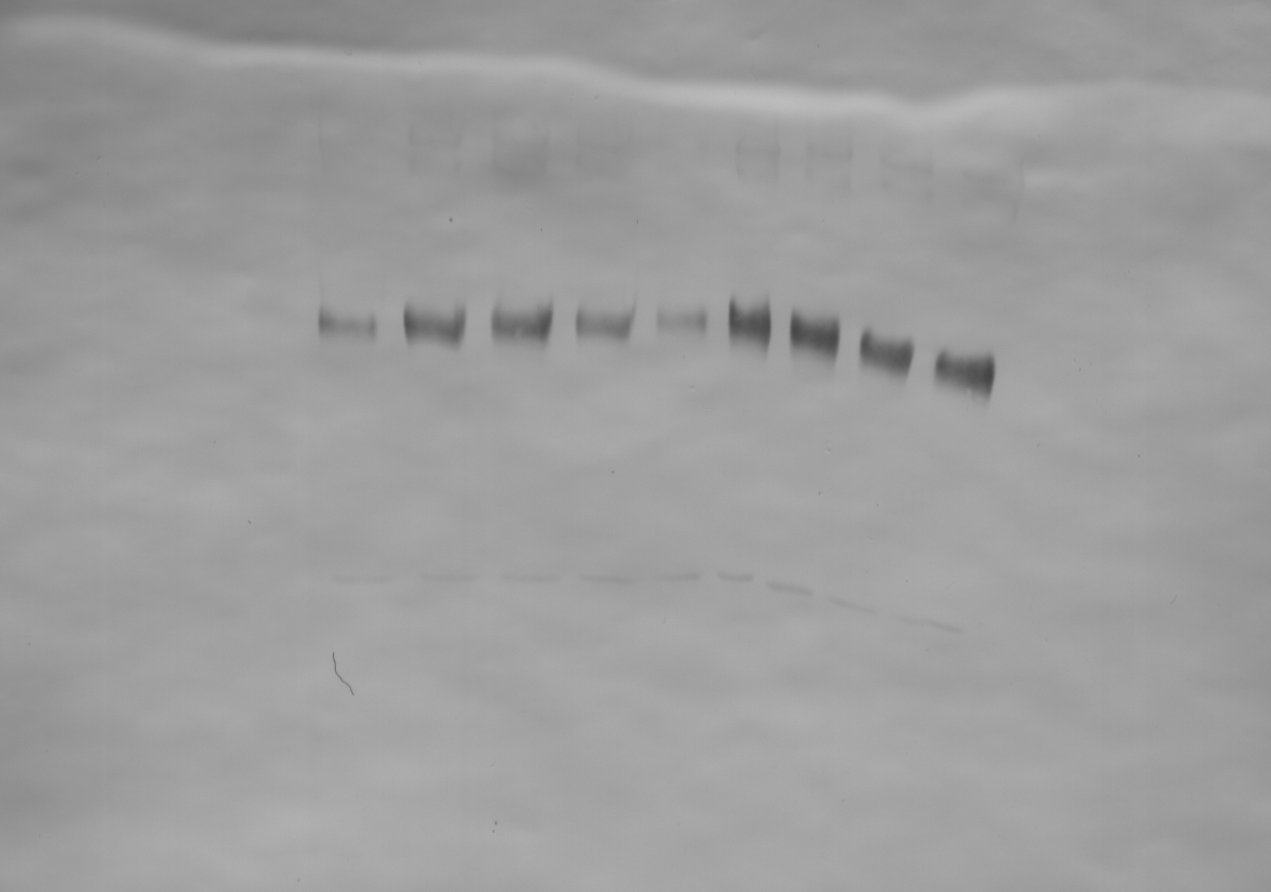

Supplement: Supplementary file 1 [file DataSheet1.ZIP › Buhrmann et al 2021-OriginalFigures-frontiers in Pharmacology/Figure 7/RKO/RKO-Bdg-assay-p-NF-kB-Original.tif]

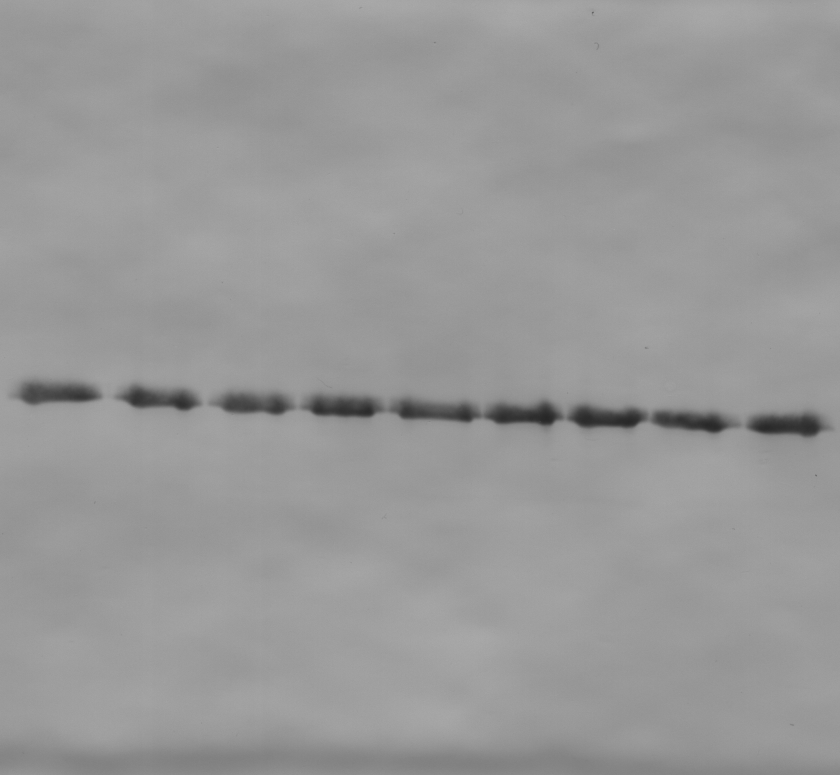

Supplement: Supplementary file 1 [file DataSheet1.ZIP › Buhrmann et al 2021-OriginalFigures-frontiers in Pharmacology/Figure 7/RKO/RKO-Bdg-assay-PARP-Original.tif]

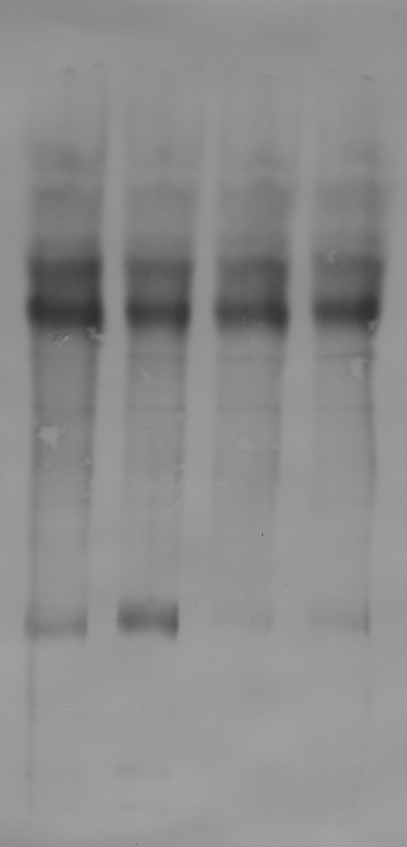

Supplement: Supplementary file 1 [file DataSheet1.ZIP › Buhrmann et al 2021-OriginalFigures-frontiers in Pharmacology/Figure 8/HCT116/HCT116-IP-NFkB-IB-Slug-Original.tif]

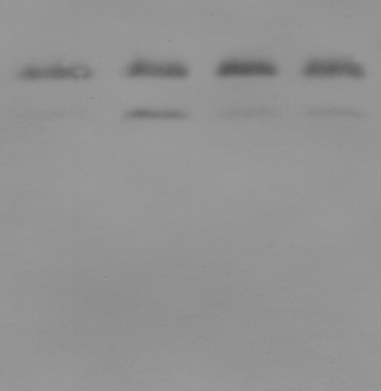

Supplement: Supplementary file 1 [file DataSheet1.ZIP › Buhrmann et al 2021-OriginalFigures-frontiers in Pharmacology/Figure 8/HCT116/HCT116-IP-Slug-WB-NF-kB-Original-ERL-Sh.tif]

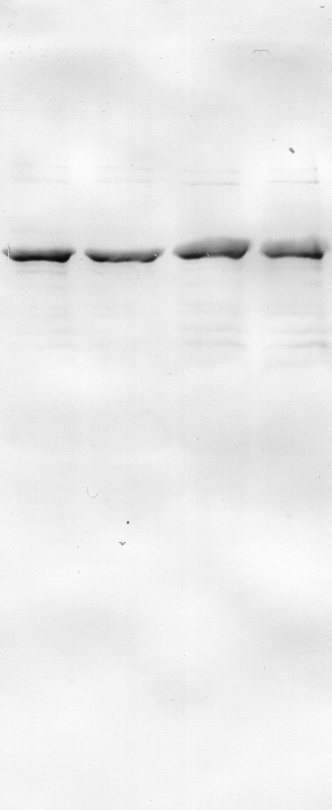

Supplement: Supplementary file 1 [file DataSheet1.ZIP › Buhrmann et al 2021-OriginalFigures-frontiers in Pharmacology/Figure 8/HCT116/HCT116-ß-Actin-IP-Original.tif]

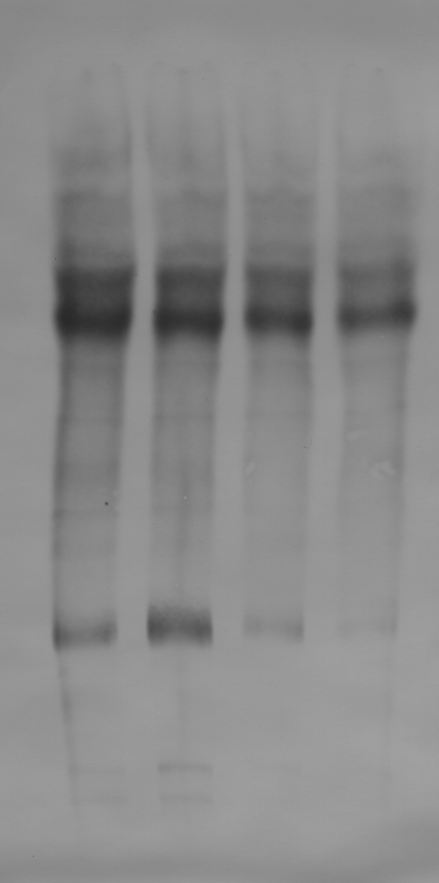

Supplement: Supplementary file 1 [file DataSheet1.ZIP › Buhrmann et al 2021-OriginalFigures-frontiers in Pharmacology/Figure 8/RKO/RKO-IP-NFkB-IB-Slug-Original.tif]

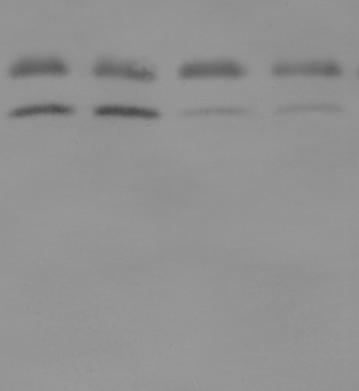

Supplement: Supplementary file 1 [file DataSheet1.ZIP › Buhrmann et al 2021-OriginalFigures-frontiers in Pharmacology/Figure 8/RKO/RKO-IP-Slug-WB-NF-kB-Original-ERL-Sh.tif]

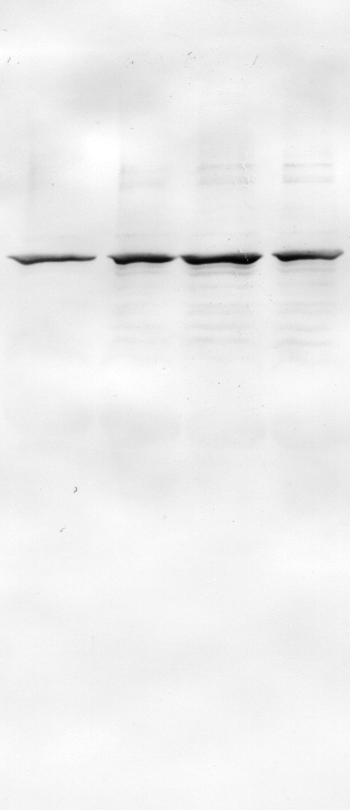

Supplement: Supplementary file 1 [file DataSheet1.ZIP › Buhrmann et al 2021-OriginalFigures-frontiers in Pharmacology/Figure 8/RKO/RKO-ß-Actin-IP-Original.tif]
